# Isotope analysis combined with DNA barcoding provide new insights into the dietary niche of khulan in the Mongolian Gobi

**DOI:** 10.1371/journal.pone.0248294

**Published:** 2021-03-29

**Authors:** Martina Burnik Šturm, Steve Smith, Oyunsaikhan Ganbaatar, Bayarbaatar Buuveibaatar, Boglarka Balint, John C. Payne, Christian C. Voigt, Petra Kaczensky

**Affiliations:** 1 Research Institute of Wildlife Ecology, University of Veterinary Medicine, Vienna, Austria; 2 Konrad-Lorenz Institute of Ethology, University of Veterinary Medicine, Vienna, Austria; 3 Great Gobi B Strictly Protected Area Administration, Takhin Tal, Gobi Altai Province, Mongolia; 4 Department of Zoology, School of Biology and Biotechnology, National University of Mongolia, Ulaanbaatar, Mongolia; 5 Wildlife Conservation Society, Mongolia Program, Ulaanbaatar, Mongolia; 6 Leibniz Institute for Zoo and Wildlife Research, Berlin, Germany; 7 Norwegian Institute for Nature Research–NINA, Trondheim, Norway; University of Hyogo, JAPAN

## Abstract

With increasing livestock numbers, competition and avoidance are increasingly shaping resource availability for wild ungulates. Shifts in the dietary niche of wild ungulates are likely and can be expected to negatively affect their fitness. The Mongolian Gobi constitutes the largest remaining refuge for several threatened ungulates, but unprecedentedly high livestock numbers are sparking growing concerns over rangeland health and impacts on threatened ungulates like the Asiatic wild ass (khulan). Previous stable isotope analysis of khulan tail hair from the Dzungarian Gobi suggested that they graze in summer but switch to a poorer mixed C3 grass / C4 shrub diet in winter, most likely in reaction to local herders and their livestock. Here we attempt to validate these findings with a different methodology, DNA metabarcoding. Further, we extend the scope of the original study to the South Gobi Region, where we expect higher proportions of low-quality browse in the khulan winter diet due to a higher human and livestock presence. Barcoding confirmed the assumptions behind the seasonal diet change observed in the Dzungarian Gobi isotope data, and new isotope analysis revealed a strong seasonal pattern and higher C4 plant intake in the South Gobi Region, in line with our expectations. However, DNA barcoding revealed C4 domination of winter diet was due to C4 grasses (rather than shrubs) for the South Gobi Region. Slight climatic differences result in regional shifts in the occurrence of C3 and C4 grasses and shrubs, which do not allow for an isotopic separation along the grazer-browser continuum over the entire Gobi. Our findings do not allow us to confirm human impacts upon dietary preferences in khulan as we lack seasonal samples from the South Gobi Region. However, these data provide novel insight into khulan diet, raise new questions about plant availability versus preference, and provide a cautionary tale about indirect analysis methods if used in isolation or extrapolated to the landscape level. Good concordance between relative read abundance of C4 genera from barcoding and proportion of C4 plants from isotope analysis adds to a growing body of evidence that barcoding is a promising quantitative tool to understand resource partitioning in ungulates.

## Introduction

Dietary niche partitioning is a key feature facilitating coexistence in multi species assemblages of wild ungulates. Competition can be reduced by partitioning plant resources according to different taxa (generalists versus specialists), parts (only leaves, fruits, buds versus the whole plant), development stages, or functional types (grass versus browse) in a shared ecosystem [1–3]. However, with ever-increasing domestic ungulate populations, competition and avoidance are increasingly shaping resource availability for wild ungulates. When resources become limiting, wild ungulates are likely to be outcompeted by domestic ungulates, as the latter often greatly outnumber the former and are favoured by human interventions [4, 5]. As a result, wild ungulates have to make do with depleted and altered rangelands or move into marginal habitats [6]. Shifts in their dietary niche are likely and ultimately can be expected to negatively affect their fitness [7]. Especially when dealing with threatened ungulate species, characterizing and monitoring the dietary niche is an important tool to detect anthropogenic impacts and guide conservation planning [8].

The wild ungulate community in Mongolia has remained almost complete, but plains species have become largely confined to the least productive or most remote parts of the country, likely in response to hunting, disturbance, and competition with livestock [9–11]. Nowadays, these remote regions, particularly the Gobi—Steppe ecosystem, constitute global refuges for several threatened ungulates, but are no longer isolated from global economic forces [12]. Mongolia’s rural economy has been based on semi-nomadic livestock husbandry for millennia and it still forms the backbone for the majority of rural communities. However, socio-political changes, with a transition from centrally planned to free-market economy, have resulted in dramatic livestock increases [13, 14]. This has led to growing concerns over rangeland health and impacts on threatened ungulates, including the Asiatic wild ass or khulan (*Equus hemionus*; [9]).

With an estimated 64,000 individuals, the Mongolian Gobi houses >80% of the global khulan population, and the situation in the Gobi determines the species’ global status and trend [15, 16]. Khulan are believed to be primarily grazers, but seem to be able to include a wide spectrum of grasses, forbs, and shrubs into their diet depending on region and season [17, 18]. However, systematic, multi-seasonal studies at the landscape scale have been scarce [19]. Sequential stable isotope analysis of wild equid tail hair from the Dzungarian Gobi (DG) in south-western Mongolia suggested that khulan primarily graze in summer, but switch to a suboptimal mixed grass / shrub diet in winter, whereas sympatric domestic horses (*Equus ferus caballus*) and reintroduced Przewalski’s horses (*Equus ferus prezewalskii*) don’t show this switch and graze year round [20].

Winter is the time of high energy expenditure for thermoregulation and in the DG coincides with the return of herders and their livestock from their summer ranges in the Altai mountains onto the wild equid range on the Gobi plains. The most likely interpretation for the switch in the khulan’s winter diet is displacement from more productive grass-dominated areas by humans and their livestock due to disturbance and persecution by humans directed at khulan, but not at charismatic Przewalski’s horses [9, 20, 21]. In line with this hypothesis, one would expect higher proportions of low-quality browse in the diet in areas where khulan share the range with livestock camps year-round, or where the density of livestock camps is higher. This is the case in the South Gobi Region (SGR) in south-eastern Mongolia, where the absence of large mountain ranges negates the benefit of seasonal altitudinal movements while proximity to urban markets makes livestock herding more profitable.

Stable isotope analysis allows differentiation between isotopically contrasted food groups, but does not provide information at the plant species level [22]. Correct interpretation of results hence requires *a priori* knowledge of the most likely food plants in a given study area [22, 23]. The plant community in the DG allows for a separation of grasses versus shrubs because C4 grasses (Poaceae, which consist of C4 as well as C3 species) are virtually absent, as they reach their northern distribution limit due to the cold and dry climatic conditions [24]. At the same time, C4 shrubs from the family Amaranthaceae persist and dominate the drier regions due to their high water retention and storage potential, deep roots, and hardy shrub lifeform [20, 24]. In the SGR, the climate is slightly warmer and moister and allows for the additional growth of C4 grasses, potentially compromising the suitability of using stable isotope analysis to assess the dietary niche of grazers along the grazing / browsing continuum.

Recent developments in DNA barcoding–taxon identification using a conserved and standardized DNA region–and massively parallel sequencing have allowed for rapid and cost-efficient analysis of large numbers of fecal samples for diet analysis at the species, genus, or at least family level [25–27]. The DNA barcoding method overcomes many of the restrictions of classical behavioural and micro-histological studies and can provide unprecedented taxonomic details of diet items in fecal samples [28, 29].

In this study, we combined isotope analysis of khulan tail hair (allowing inferences about multi-year diet seasonality and isotopic dietary niches) and fecal samples (providing a snapshot of the diet over the last 1–2 days; [30]) with DNA barcoding of fecal samples (allowing accurate plant taxa identification). Our overall goal was to achieve a more comprehensive understanding of the diet switch previously observed during the winter energetic bottleneck in one of two study areas in the Mongolian Gobi. Our two primary aims were to: i) verify previous results and assess whether the same analytical framework can be used for ungulates throughout the Gobi region and ii) look for support that the diet shift in winter in the DG is linked to seasonal differences in degrees of human and livestock presence. Specifically, we expected to find:

1. Based on stable isotope analysis of tail-hair samples–a seasonal shift from a grass dominated C3 summer diet to a shrub dominated C4 winter diet in the SGR in line with previous results from the DG;
2. Based on the DNA barcoding results–confirmation of the switch from a grass dominated summer diet to a mixed grass/browse diet in winter via taxonomic identification of plant remains in winter feces from the DG, but a less clear separation in winter feces from the SGR due to a climate less exclusive to C4 grasses;
3. Based on a comparison of both methods for fecal samples–high consistency between isotope signatures and DNA barcoding in respect to the proportion of C3/C4 plants identified in the diet;
4. Based on combined results from both methods–higher levels of C4 shrubs in the diet of khulan in the SGR due to the higher density and year-round presence of herder camps and the resulting higher potential for displacement by and competition with humans and their livestock.

We believe this study provides a valuable framework for future studies of ungulates in drylands and provides important empirical insights into the potentials and limitations of dietary analysis methods which are relevant for future research in general, and ungulate conservation in the Gobi specifically.

## Materials and methods

### Study area

**Fig 1:**
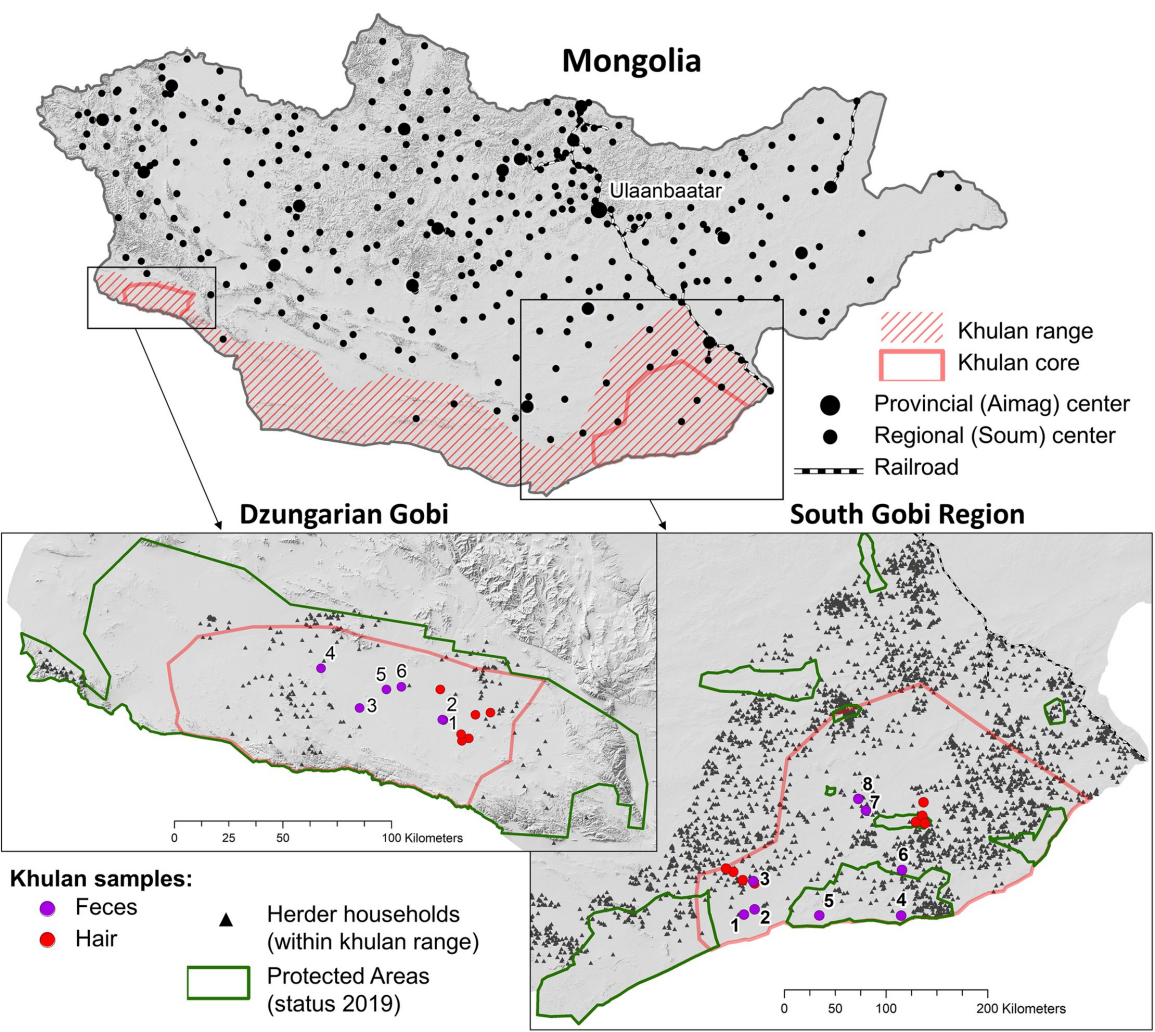
The two study areas in the Mongolian Gobi. The ca. 9,000 km^2^ khulan distribution core in the Dzungarian Gobi (DG) does not include any villages and no herder camps are present in summer. The ca. 56,000 km^2^ khulan distribution core in the South Gobi Region (SGR) contain several settlements and herder camps are present year-round. Figure generated in ArcGIS 10.7.1 (ESRI, Redland, CA, USA, http://www.esri.com/) using NASA SRTM Digital Elevation 30m data for background.

The two study areas are located at the eastern and western end of the khulan distribution in the Mongolian Gobi (Fig 1). Towards the south and west, the khulan distribution range in the Gobi is limited by the fenced international border towards China, towards the east by the fenced Trans-Mongolian railway, and towards the north by increasing human and livestock density [31]. The western study area in the DG covers roughly 9,000 km^2^ and falls entirely within Great Gobi B Strictly Protected Area which was extended from 9,000 km^2^ to 18,000 km^2^ in 2019. The eastern study area covers roughly 56,000 km^2^ in the SGR. Both study areas fall primarily in the semi-desert zone, but vary somewhat in respect to topography, climate, vegetation, khulan population size, and lands use (Table 1, S1 File).

**Table 1:** Characterization of the two study areas at the western and eastern end of the khulan range in the Mongolian Gobi.

Habitat parameter	Dzungarian Gobi (DG)	South Gobi Region (SGR)	References
Latitude	45^o^	43^o^	
Terrestrial Ecoregions of the World (TEOW)	Dzungarian basin semi-desert	Alashan plateau semi-desert & Eastern Gobi desert steppe	[32]
Size of study area	9,000 km^2^	56,000 km^2^	
Area within national protected areas	100% [Great Gobi B Strictly Protected Area]	17%	
Landscape type	Large plains, rolling hills, and small mountain ranges flanked by high mountains towards the S, E, W	Large plains, interspersed with hills and low mountain ranges	
Range in elevation	1,000 to 2,900m	680 to 1,900m	
Average annual temperature	2°C	7°C	
Range in monthly temperature averages	-18°C to 18°C	-13.4°C to 24.9°C	
Average rainfall	100mm	100mm to 150mm [increase from southwest to the northeast]	[33]
Average snowcover	100 days	irregular	
Dominant plant families	Amaranthaceae (formerly Chenopodiaceae), Poaceae (grasses), Asteraceae, and Tamaricaceae	Same as DG	DG & SGR: [33] DG: [34], also see S1
Dominant plant communities	*Haloxylon ammodendron* associations (39% coverage) and grass-dominated *Stipa* associations (35% coverage)	More diverse, coverage data not available	
Expected presence of C4 plants	Dominant grasses and forbs: C3; many shrubs and semi-shrubs, particularly Amaranthacaeae: C4	As DG, but additional C4 grasses like *Cleistogenes songorica*, *Eragrostis minor* and *Aristida heymannii* are found amid the dominating *Stipa* (C3) communities	[20, 24, 33, 35]
Herder presence / year	ca. 9 months	12 months	
Seasonality of herder presence	Spring, fall, winter [altitudinal migration to alpine pastures in the Altai mountains in summer]	Year-round [no altitudinal migration possible due to lack of large mountain ranges]	
Herder camp density	0.01 / km^2^	0.02 / km^2^	Statistical Office of Mongolia 2018
Average livestock density (all species)	6.96 / km^2^	10.25 / km^2^	
Average sheep food units (SFU) [36]	9.42 / km^2^	17.61 / km^2^	
Two most recent khulan population size estimates	2010: 5,671 (95% CI = 3,611–8,907)	2015: 36,298 (95% CI = 21,447–61,434)	DG: [37], Kaczensky et al. unpubl. Data
	2015: 9,337 (95% CI = 5,337‐16,334)	2019: 51,691 (95% CI = 33,658–79,386)	SGR: [16], B. Buuveibaatar unpubl. data
Survey area	11,000 km^2^	98,000 km^2^	
Estimated khulan population density	0.85 km^2^	0.53 km^2^	
Human infrastructure	None	Several villages, mine sites and associated infrastructure	[12, 38]

### Sample collection and preparation

For multi-year diet analysis, we plucked several long tail hairs from adult khulan during capture and anaesthesia when GPS collars where attached to khulan as part of ongoing monitoring programs. For analysis, we selected individuals with the longest tail hairs for maximal temporal coverage, namely 3 females and 3 males captured in July 2009 in the DG (Fig 1, S2 Table; [20]) and 4 females and 4 males captured in August 2013 in the SGR. Hair samples were stored in labelled paper envelopes until arrival in the laboratory where they were cleaned and cut into 1 cm increments for sequential CN or H analysis, as reported in [39].

For winter diet analysis, we collected fresh fecal samples from khulan in February 2016: 42 samples at 6 different locations in the DG and 25 samples at 8 locations in the SGR (Fig 1, S3 Table). Samples were collected in plastic bags and kept cool before and during transport to the laboratory where outer layers were carefully removed to avoid contamination with other DNA caused by contact with the environment and to reduce endogenous DNA proportions. The remaining material was dried to constant weight at 50° C and ground using a mortar and pestle for isotope analysis and barcoding.

Hair and fecal samples were imported to Austria in accordance with the Convention on International Trade in Endangered Species of Wild Fauna and Flora (CITES, no. AT 15–0992) and respective veterinary permits (no. BMG-74130/0110-II/B/10/2015 and BMG-74130/0026-II/B/10/2016). Khulan captures was authorized by the Mongolian Ministry of Nature, Environment and Tourism and the ethic commission at the University of Veterinary Medicine Vienna was informed and provided general consent (ETK-15/03/2016).

### Stable isotope analysis

The stable isotope analysis was conducted at the Leibniz Institute for Zoo and Wildlife Research, Berlin, Germany following methods described in ([20, 39] and S1 File). The precision of measurements was always better than 0.1 ‰ for *δ*^13^C and *δ*^15^N, and 1.0 ‰ for non-exchangeable *δ*^2^H values, based on repeated analysis of laboratory standards, calibrated with international standards. In the context of this study, *δ*^2^H values were primarily used to assign the *δ*^13^C profiles to the correct seasons [39].

To convert raw hair isotope values (*δ*^13^C_hair_ and *δ*^15^N_hair_) into diet values (*δ*^13^C_diet_ and *δ*^15^N_diet_), we used the dual mixing model of [40]. To calculate the % of C4 diet in the feces, we used a dual end-member mixing model from [41], where % C4 = (*δ*^13^C_C3 plants_ + Δ*δ*^13^C - *δ*^13^C_feces_) / (*δ*^13^C_C3 plants_ -Δ*δ*^13^C_C4 plants_).

We used a Bayesian approach to estimate species specific core isotopic dietary niches based on bivariate, ellipse–based metrics (for *δ* ^13^C and *δ* ^15^N values) using SIBER implemented in the R package SIAR (Version 4.2); see S1 File for more details on plant sampling, stable isotope analysis, and visualization.

### DNA analysis of fecal samples, species identification and plant metabarcoding

#### DNA extraction and amplification

Total genomic DNA was extracted from 100 mg of powdered fecal material using the FastStool kit (Qiagen, Germany) according to the manufacturer’s instructions but with some slight modifications. Specifically, the incubation with Buffer AL was performed at 95° C (rather than 70° C) for 10 minute and the final column elution step was carried out in 2 x 20 μl volumes for 10 minutes each. Extracts were stored at -20°C until required for downstream analyses.

Dietary NGS library preparation followed the protocol of [25] using three primer sets to broadly cover all plant orders (trnL(UAA)g-h) while providing enough taxonomic resolution to differentiate genera from Asteraceae (ITS1-Ast) and from Poaceae (ITS1-Poa; S4 Table). For details on PCR and library purification and pooling (S2 File).

#### Sequencing and data analysis

Sequencing was performed as 125 basepair paired end (PE) on an Illumina HiSeq 2000 at the Vienna Biocenter Core Facility. Data were delivered as BAM files and converted to fastq format using the bam2fq command in SAMtools [42]. Demultiplexing was performed with the command barcode_splitter (FastX toolkit, Hannon Lab). Paired reads were merged using the tool FLASH with the “innie” and “outie” options implemented to ensure that short reads that include sequencing adaptors are also incorporated. Sequencing adaptors and primers were then removed using the tool Flexbar before quality filtering to remove merged reads with greater than 20% of bases with quality scores less than Q30 (FastX toolkit, Hannon Lab). We also included two small experiments, sequencing 50:50 mixes of the five most important plants from the DG to test for amplification bias (S1 Fig) and running a 2^nd^ subsample for six feces from the SGR to get a feeling for within-fecal sample variation (S2 Fig).

Reads were processed using the UParse pipeline [43] to create clusters of operational taxonomic units (OTUs) showing >97% similarity while simultaneously removing chimeric sequencing artefacts (all relevant scripts can be found in S3 File). Taxa was assigned to OTUs by blasting sequences against the NCBI Viridiplantae sequence database within the program CLC-Main Workbench (Qiagen, Germany). Settings were an Expect value of 10.0, Word size of 11, Match value 2, Mismatch value -3, gap costs of Existence 5 and Extension 2. Maximum number of hit sequences was set to 2. Taxonomy was accepted only if identity was >95% and abundance was greater than 0.01% overall. All species sequence reads were collapsed down to genus level.

We were not able to collect and sequence all potential plant species or genera over the huge expanse of the two study areas and did not expect all plant genera to be available in the NCBI Viridiplantae sequence database. Therefore, rather than *a priori* removing genera not native to Mongolia or the Gobi and potentially introducing a bias towards better known plant genera, we used a sequence read cut-off value of 0.5% over all genera reads by region (for read abundance RA) and individual scat (for frequency of occurrence FOO). The remaining sequence reads were checked for their presence in Mongolia and the Gobi based on (S5 Table). Our cut-off value removed all but one genus not found in Mongolia/Asia and two without documented records from the Gobi; all three were only of minor importance. We assume that there must be an unidentified closely related species in our study area or a misclassification sequence in the database [44]. Whether or not we included these three genera made little difference to the overall results; the chose to include them which gave a slightly higher importance and diversity to the Asteraceae family.

The method of calculating diet proportions influences the results of diet characterization. Initial studies suggest that using RA introduces biases particularly for common diet taxa [45] while using absolute or relative FOO of taxonomic units is sensitive to small samples sizes and likely overestimates the importance of rare items or abundant species accidentally ingested together with the target species [46]. To allow comparison with previous studies we chose to present diet for each region at the genus level as: 1) RA, representing the proportion of sequence reads belonging to a unique genus in a fecal sample, divided by the total number of sequence reads in that fecal sample and 2) FOO, representing the number or percentage of feces in which a unique sequence read for a genus was found with a frequency >0.005. We additionally derived relative frequency of occurrence (rFOO) by dividing the sum of the FOO of each genus by the sum of the FOO of all genera (and families for comparison with [47]). We used the Bray-Curtis index, scaled from 0 (complete diet overlap) to 1 (no diet overlap), calculated with *vegdist* in the R package vegan [48] to compare diet dissimilarities among samples and sampling sites.

## Results

### Seasonal isotope pattern of khulan tail hair and feces

**Fig 2:**
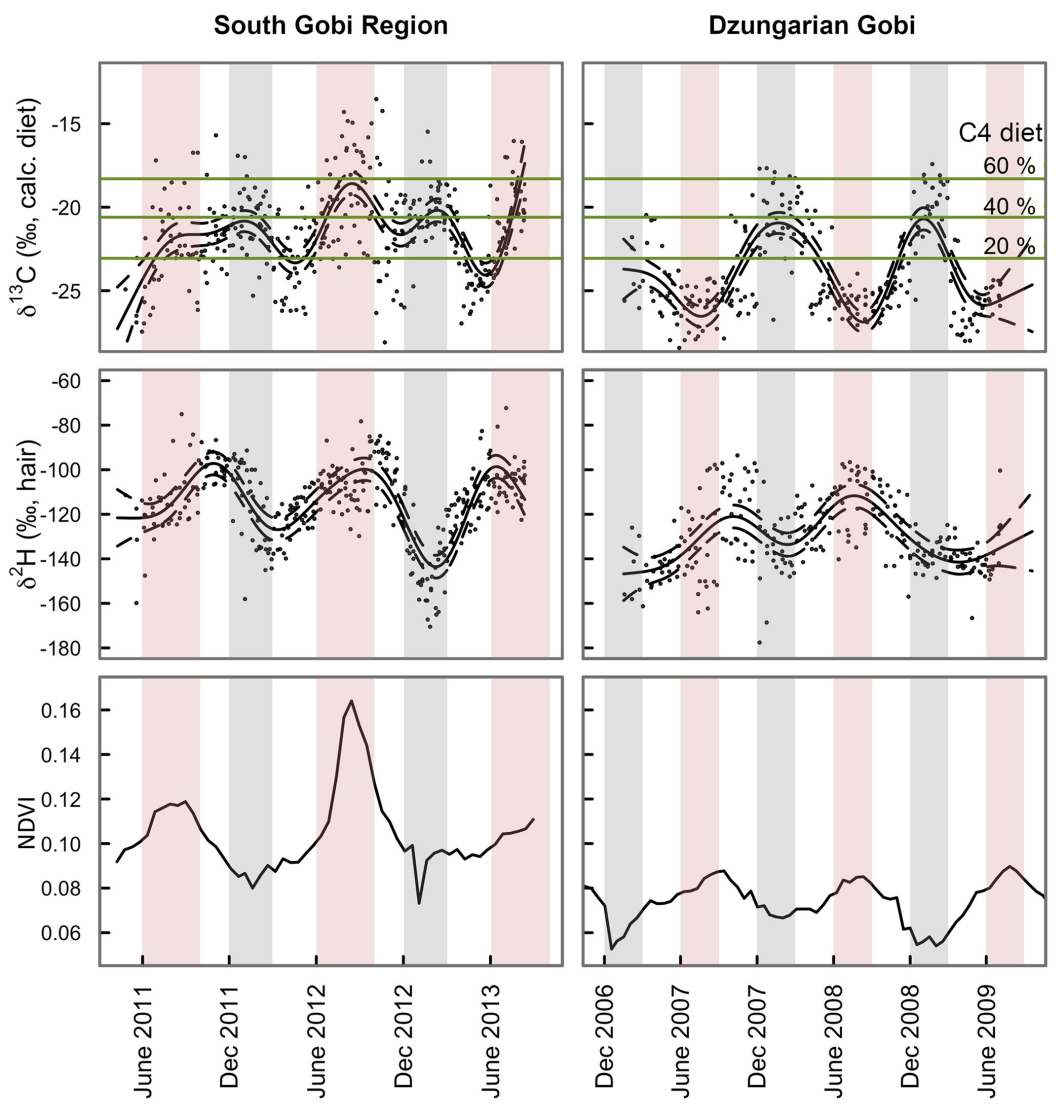
Isotope profiles of khulan tail hair. Sequential, temporally explicit ™^13^C_diet_, ™^2^H_hair_ profiles of khulan tail hair and time-matched 16-day NDVI values. The fraction of C4 biomass in the diets takes into account the isotopic variability of the C3 and C4 end members used in the mixing model (mean ™^13^C ± 1σ, depicted with solid and dashed line, respectively). Pink and gray stripes depict summer and winter, respectively.

Isotope values of khulan tail hairs from the SGR did not show the expected diet switch from high *δ*^*13*^*C*_*diet*_ values in winter (up to 67% C4 plant intake) and low values (<10%) in summer as previously shown for the DG in [20]. Rather, values followed a bimodal pattern with highest *δ*^*13*^*C*_*diet*_ values (high C4 plant intake of up to 84%) in mid-summer and mid-winter and lower values in fall (<40%) and spring (<20%; Fig 2).

Isotope values of khulan feces from winter showed only a slightly higher mean intake of C4 plants in the SGR (40.9%) as compared to the DG (37.5%, Table 2). The core winter isotopic dietary niches of hair segments and fecal samples were very similar, showing a mixed C3/C4 diet for both regions and types of samples (S3 Fig).

**Table 2:** Relative consumption of C4 plants of khulan in winter from two different regions in the Mongolian Gobi based on different sample types and analysis methods.

C4 plants diet in winter based on different methods		Dzungarian Gobi (%)	South Gobi Region (%)
Tail hair SI	Mean	**38.5**	**42.6**
	Min	15	4.5
	Max	71	85
	N*	6(76)	8(85)
Feces SI	Mean	**37.5**	**40.9**
	Min	0	0
	Max	67	83
	N	42	25
Feces barcoding	Mean RA	**20.1**	**42.6**
	Min RA	0.9	1.9
	Max RA	42.1	94.7
	Mean FOO	**31.0**	**42.2**
	N	42	24

RA = abundance reads, FOO = frequency of occurrence.

*Number of individuals sampled with their cumulative number of winter tail hair segments in brackets.

### Winter diet based on barcoding of feces

**Fig 3:**
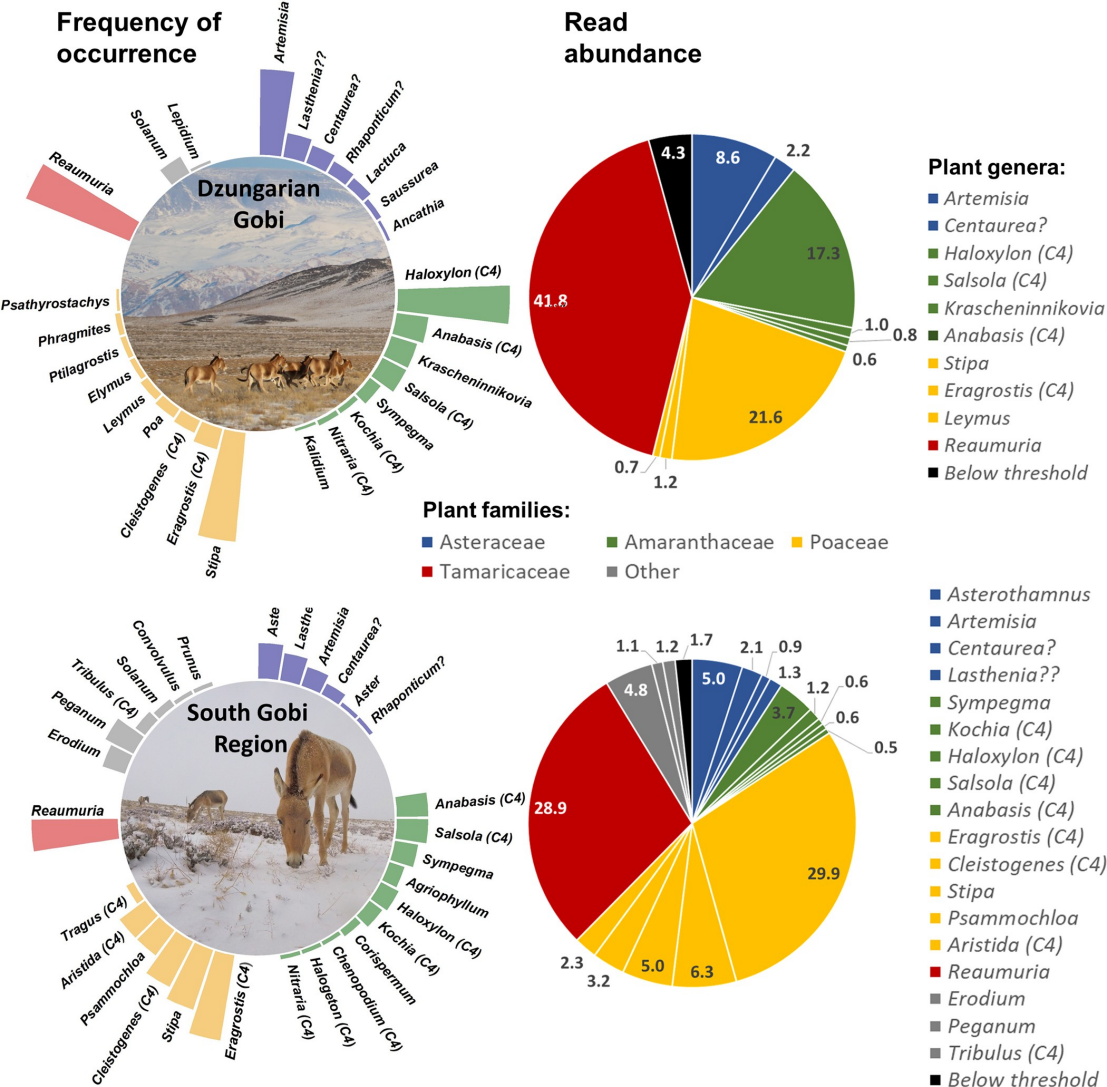
Khulan winter diet based on barcoding. Frequency of occurrence and relative read abundance of plant genera identified by barcoding in 66 kulan fecal samples (42 from Dzungarian Gobi and 24 from the South Gobi Region) from winter in the two study areas in the Mongolian Gobi. Genera appear clockwise in the pie diagram in the order they appear in the legend.? = 2 genera not recorded for the Gobi and?? = 1 genera not recorded for Mongolia (see methods for details). Photos: Dzungarian Gobi N. Altansukh, South Gobi Region: Camera collar.

Using FOO, we identified 39 genera from 11 families, of which 27 genera from 6 families were present in feces from the DG and 29 genera from 10 families in feces from the SGR (Fig 3, S5 Table). Using RA the number of genera was reduced to 10 genera from 4 families for the DG (representing 96% of all reads) and 18 genera from 7 families for the SGR (representing 98% of all reads; Fig 3).

**Fig 4:**
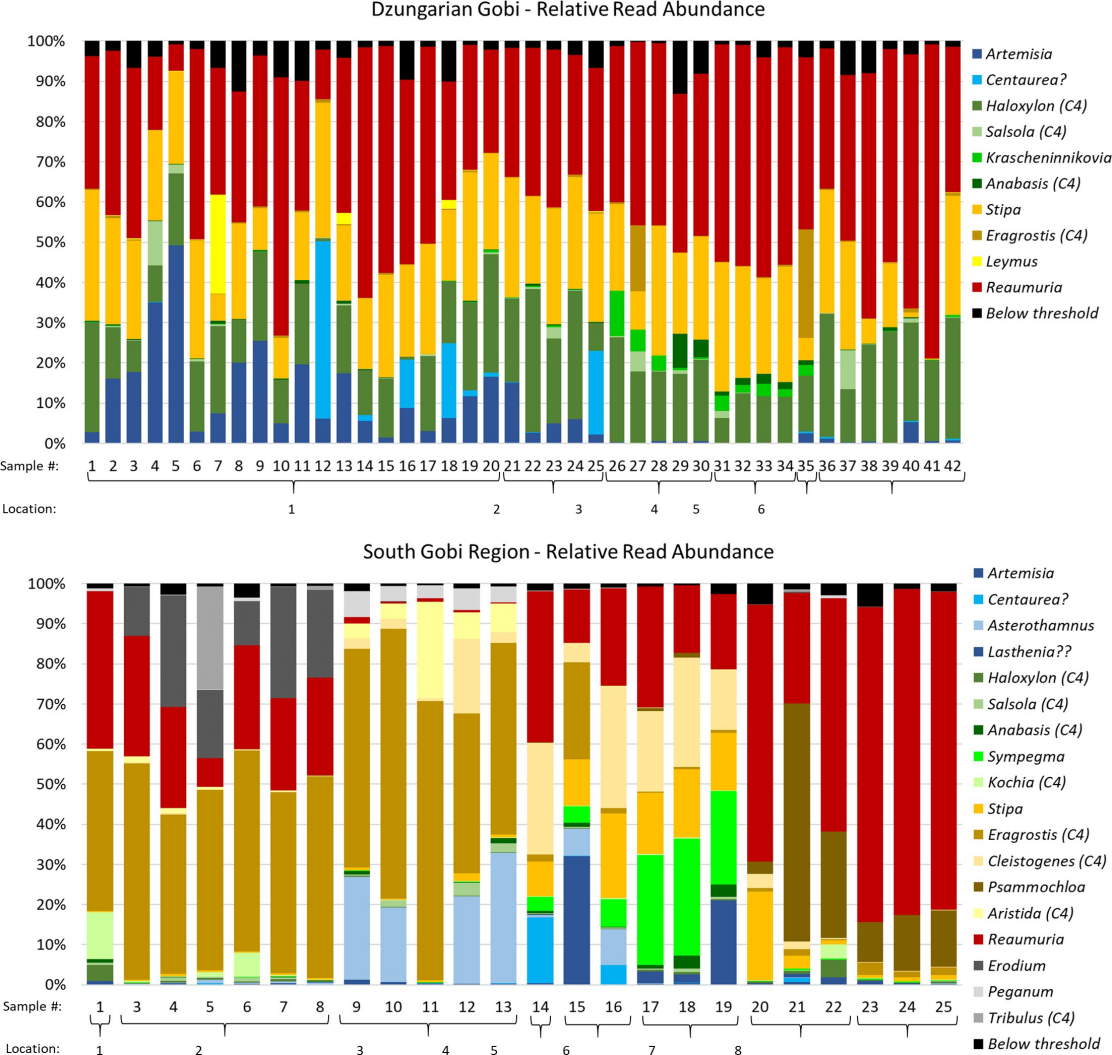
Diet similarity of individual fecal samples. The comparison is based on read abundance of plant genera (>0.5% of all sequence reads in each sample) in 66 kulan feces from winter in the two study area in the Mongolian Gobi. Genera with color codes in the legend show the order in which they appear in the bar, from bottom to top. Color ranges codes match family colors in previous figures: blue = Asteraceae, green = Amaranthaceae, orange = Poaceae, red = Tamaricaceae, grey = remaining rare families (Geraniaceae, Peganiaceae, *Zygophyllaceae)*, black: sum of all remaining genera reads which fell under the <0.5% threshold (for details see method section). Location = sample location (see Fig 1 for map).

Diet calculations based on FOO and RA resulted in different proportions given to diet items at the genera level but agreed on the bigger picture. Poaceae, Amaranthaceae, Tamaricaceae, and Asteraceae were identified as the most important plant families in the winter diet of khulan in the DG and the SGR. The importance of Tamaricaceae (represented in the diet by the single genus Reaumuria with one species in the Gobi) is undisputed but may be somewhat overestimated because of an amplification bias in favour of the Reaumuria (for details see S1 Fig). Six additional plant families were of minor importance, with four only found in the SGR and one only found in the DG (Figs 3, 4, S5 Table).

The diet in the SGR was more diverse and, contrary to our expectation, it was dominated by Poaceae, while the diet from the DG was a more even mix of Amaranthaceae, Poaceae, Tamaricaceae, and Asteraceae. *Reaumuria*, *Stipa* (Poaceae), and *Haloxylon* (Amaranthaceae) were the most important genera in the DG and *Eragrostis* (Poaceae) and *Reaumuria* the most important genera in the SGR (Fig 3).

Genera with a C4 photosynthetic pathway were more important in the SGR as compared to the DG (Table 3, Fig 3). However, contrary to our expectation, C4 genera in the SGR were primarily recruited from Poaceae rather than from Amaranthaceae like in the DG. In general, FOO suggested a higher degree of similarity in diet composition between the two study areas than RA (Table 3, Fig 3).

**Table 3:** Percentage representation of all genera and those with a C4 photosynthetic pathway identified with barcoding combined by family.

Family	Dzungarian Gobi			South Gobi Region		
	% RA	% rFOO^1^ [Fig 4]	%rFOO^2^	% RA	% rFOO^1^ [Fig 4]	%rFOO^2^
***Plant family representation***						
Asteraceae	10.7	22.9	19.4	9.2	14.6	15.5
Amaranthaceae	19.7	32.1	25.5	6.6	22.5	19.4
Poaceae	23.5	26.0	24.8	46.7	38.8	23.3
Tamaricaceae	41.8	16.0	25.5	28.9	12.4	21.4
Other	4.3	3.1	4.8	8.7	11.7	20.4
***C4 plant representation***						
Amaranthaceae	18.9	26.0	77.8	2.9	15.2	38.1
Poaceae	1.2	5.0	22.2	38.5	25.3	54.8
Other	0.0	0.0	0.0	1.2	1.7	7.1
**All C4 combined**	**20.1**	**31.0**	**100.0**	**42.6**	**42.2**	**100.0**

For comparison with other studies, we provide read abundance (RA) over all feces (cut-off value of 0.5% over all samples) versus relative frequency of occurrence (FOO) in individual feces (cut-off value of 0.5% for individual samples to define occurrence).

^1^Sum of rFOO calculated by genera

^2^Recalculted at family level for comparison with [47], see discussion.

Despite a larger number of genera found in the feces, diet dissimilarities among feces based on RA were only slightly higher in the SGR as compared to the DG (mean Bray-Curtis dissimilarity = 0.35 and 0.31 respectively; low values = high overlap or low dissimilarity). Feces were more similar within sampling location than among all locations pooled, with the effect being stronger in the SGR as compared to the DG (weighted mean Bray-Curtis dissimilarity = 0.11 and 0.25 respectively; Fig 4).

There was an overall good agreement in the proportions of C4 plants in the winter diet of khulan based on isotope analysis (tail hair and feces) and barcoding (feces) with values being consistently higher for the SGR as compared to the DG (Table 2).

## Discussion

### Concordance of barcoding results with past isotope analysis

Our barcoding results revealed an unexpected diet composition in the SGR, but they also largely confirmed the interpretation of previous isotope analysis of tail hair from the DG [20]. Khulan in the DG feed on a mixed grass / shrub diet in winter, the consumed grasses are largely composed of C3 grasses from the genus *Stipa*, and a large proportion of consumed shrubs is composed of C4 shrubs, namely *Haloxylon ammodendrum*, *Anabasis brevifolia*, and *Salsola* from the family Amaranthaceae. New insights highlight the importance of genera in the Asteraceae family, providing further evidence that the importance of forbs may have been underestimated with conventional diet analysis, at least for equids living under semi-desert or desert conditions [49].

Genera identified with barcoding for the DG roughly matched results of micro-histological analysis by Xu et al. [9] in respect to the high importance of *Stipa*, *Haloxylon*, and *Reaumuria*, but differed greatly in respect to *Anabasis* (S6 Table). For rarer genera, differences vary considerably, and interestingly, [19] failed to detect any C4 grasses, which may point to the difficulty of finding rare diet items or distinguishing between anatomically similar species [29]. On the other hand, feces only provide a snapshot of a khulan’s diet over the last 1–2 days [30] and fecal composition varies within and, even more so, between sample locations. Given the relatively small sample sizes of studies available so far and the method differences between barcoding and micro-histological studies, discrepancies for diet items of lower importance are hardly surprising and should not be overinterpreted [29, 50].

### Khulan diet seasonality in the South Gobi Region

At first glance, our isotope data seems to support the hypothesis that khulan in the SGR are forced to shift to a more C4 browse-dominated diet due to the higher density and year-round presence of herder camps [20]. The isotope analysis confirmed higher levels of C4 plants and identified a bi-modal seasonal diet shift in the SGR with C4 peaks in winter and summer. However, subsequent barcoding contested the validity of this conclusion as it identified C4 grasses as the main source of C4 plants in the winter diet of khulan in the SGR. As it turns out, genera with C4 grasses like *Eragrostis*, *Cleistogenes*, and *Aristidia* dominated the winter diet, rather than only complementing the consumption of the widespread and common C3 *Stipa* grass, which constitutes a staple for khulan in the DG. Furthermore, the shrubs consumed were composed of genera that included C4 and C3 species instead of being dominated by C4 species as in the DG. This mix of genera with C3 and C4 pathways in grasses and shrubs makes the unambiguous isotopic separation along the grazer—browser spectrum impossible for the SGR.

Barcoding allowed us to unambiguously identify C4 grasses as the main source of C4 plants in the diet of khulan in the SGR in winter. We currently lack this information for the C4 summer peak, though C4 grasses seem the most likely candidate [47]. The C3 lows in spring are likely a consequence of the first emergence of C3 spring forbs like *Allium* and cold robust C3 grasses before increasing temperatures allow the re-emergence of C4 grasses [51]. The same may be happening with C3 and C4 grasses in fall, with the former being photosynthetically active longer than the latter and therefore preferred by grazers like khulan. However, additional seasonal studies are required to confirm an overall, year-round high use of C4 grasses. Moreover, if confirmed, further field investigations will be needed to test whether the high C4 grass use reflects their higher availability in areas where khulan roam or is the result of a preference over common C3 grasses like *Stipa*.

### Unresolved question of diet shift in response to humans

Climatic and biogeographic changes from the western to the eastern Gobi result in such profound diet differences in khulan that predictions from one region cannot be tested in the other region within a simple comparative framework. Our results, highlighting a grass-dominated winter diet in the SGR, offers the tantalising assumption that this occurs despite disturbance by and competition with local herders and their livestock, and that one would expect that the summer diet in the SGR consists entirely of abundant grasses and forbs and excludes shrubs altogether. Unfortunately, we lack the seasonal barcoding data to confirm this hypothesis. Barcoding results from khulan summer feces, collected and analysed by [47] suggests that use of the shrub dominated plant families Amaranthaceae and Tamaricaceae is reduced, use of Poaceae remains high, and use of forbs increases and diversifies. The summer diet includes several new families with genera typical for spring and summer and absent in winter like *Alium* (Amaryllidaceae) and *Iris* (Iridaceae; S4 Fig). However, while there is some evidence of a diet shift in khulan from summer to winter, quantitative comparisons with our results are not possible because [47] used the trnL primer alone which results in a lower taxonomic resolution. Re-calculating diet composition for comparison as FOO at the family level, results in a great loss of taxonomic detail and hence is unsuitable for detecting more subtle differences or gradual shifts in diet.

With 23–47% (depending on study area and method of calculation) the proportion of grasses in the winter diet of khulan in the Mongolian Gobi is generally low compared to other wild and free-ranging equids for which grasses are the primary diet items, typically amounting to 70–100% [18, 25, 49]. However, without a fully comparative framework which includes sympatric domestic species and nutritional analysis, it is difficult to draw inferences about whether these changes solely reflect seasonal availability of preferred plants or point towards competition with, or displacement by, livestock.

### Methodological considerations

Our barcoding results showed good consistency with isotope analysis in respect to the proportion of C4 plants in the diet and also with the micro-histological study by [19]. Our study therefore adds to a growing body of evidence that barcoding and the use of RA is a promising quantitative tool, especially for comparative diet studies within the same bio-geographical region, where the main interest is diet dissimilarity or diet trends, rather than diet composition *per se* [25, 46]. The latter is more difficult to obtain as results are influenced by primer selection, amplification bias (as shown also in our own small experiment), potential interactions with other food items, differences in the density of target DNA among plants and between different structural parts of the same plant, and different sensibility of plant matter to digestive breakdown [28, 50]. However, alternative methods have their own challenges [29].

The comparative nature of our study and the use of an identical analysis framework accounts for most of the aforementioned constraints. We are therefore confident that our results allow for a valid comparison of the winter diets between the two regions of the Mongolian Gobi and provide sufficient taxonomic resolution for a more comprehensive understanding of the diet switch previously observed during the winter energetic bottleneck in the DZ. In addition, we identified a large spectrum of genera utilized by khulan, several of which had not been previously identified. The unexpected high use of C4 grasses in the SGR raises the question of whether khulan, which are generally considered bulk grazers, may actually be much more selective than previously assumed.

## Conclusions

Our analysis approach of combining isotope analysis of tail hair and barcoding of feces 1) provided unprecedented taxonomic detail and documented previously unknown differences in the winter diet of khulan in two different parts of the Mongolian Gobi, 2) established that longitudinal isotope analysis of long tail hair in an ungulate is a suitable tool to detect and compare population level diet shifts, 3) confirmed that the specific climatic conditions in the DZ allow for an isotopic differentiation along the grazer-browser continuum, and 4) revealed that the same conditions are not met in the SGR.

Our results once again highlight the importance of parallel analysis approaches when using indirect analysis methods, especially when moving from intensively studied to less intensively studied or larger study areas. The fact that khulan feces were more similar within sampling locations than among sampling location, highlights: 1) the need for fecal sampling to be spread across a region to account for the influence of landscape heterogeneity, 2) a potential for using the habitat characteristics of the wider sampling location as covariates to explain diet differences.

## Supporting information

S1 Fig Amplification bias. (DOCX)

S2 Fig Abundance read variability. (DOCX)

S3 Fig Core isotopic niche areas. (DOCX)

S4 Fig Comparison of results with Sugimoto et al. 2018. (DOCX)

S1 Table Main habitats and most common plants. (DOCX)

S2 Table Khulan tail hair samples. (DOCX)

S3 Table Khulan fecal samples. (DOCX)

S4 Table Primers for DNA barcoding. (DOCX)

S5 Table Plant genera identified with DNA barcoding. (DOCX)

S6 Table Comparison of results with Xu et al. [9]. (DOCX)

S1 File Stable isotope analysis details. (DOCX)

S2 File DNA barcoding analysis details. (DOCX)

S3 File Analysis pipeline and scripts for the metabarcoding analysis. (ZIP)

S1 Graphical abstract (TIF)
