# Supplementary material for: Isotope analysis combined with DNA barcoding provide new insights into the dietary niche of khulan in the Mongolian Gobi
Source: PLoS One. 2021 Mar 29;16(3):e0248294. doi: 10.1371/journal.pone.0248294 (PMC8006982; doi:10.1371/journal.pone.0248294)
Supplement: S1 Fig — (DOCX) [file pone.0248294.s001.docx]

## S1 Fig. Amplification bias.

To test for amplification bias, we ran a small experiment mixing equal amounts of the 5 most common genera from the Dzungarian Gobi in in equal proportions by weight. Barcoding results did identify both genera in all four mixtures but showed a deviation from the expected 50:50 amplification with *Reaumuria* occurring at >50% in all four combinations. The percentage of unassigned genera was higher in mixtures with genera from the Amaranthacaeae (*Haloxylon* & *Anabasis*) as compared to those from Poacea (*Stipa*) and Asteraceae (*Artemisia*) for which a family specific primer was used (ITS1-Ast and ITS1-Poa; Fig. S7).


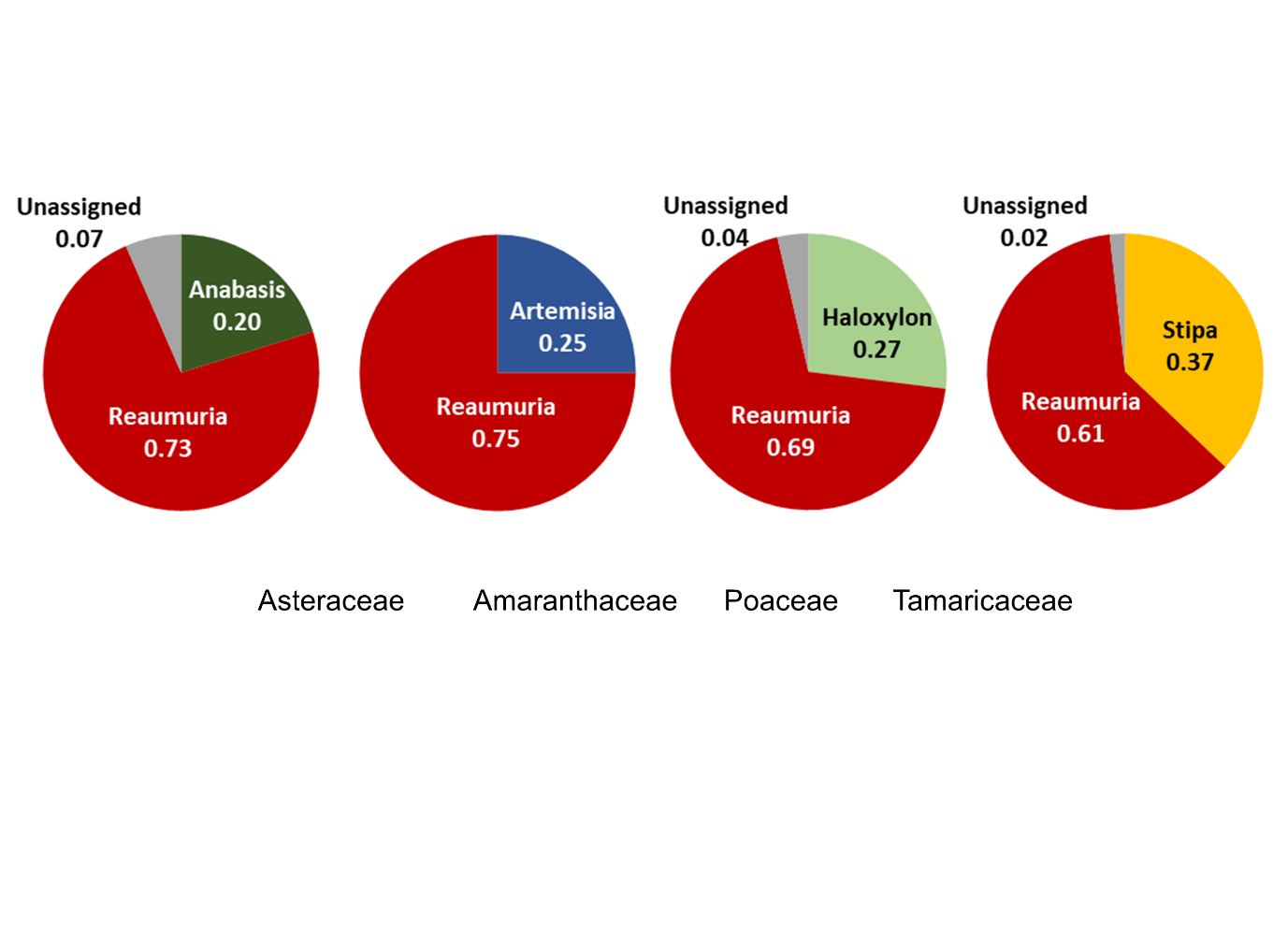


**S1 Fig**. Relative read abundance of four mixtures of equal proportions (powdered plant material by weight) of the five most common plant genera from the Dzungarian Gobi.
