# Supplementary material for: Isotope analysis combined with DNA barcoding provide new insights into the dietary niche of khulan in the Mongolian Gobi
Source: PLoS One. 2021 Mar 29;16(3):e0248294. doi: 10.1371/journal.pone.0248294 (PMC8006982; doi:10.1371/journal.pone.0248294)
Supplement: S2 Fig — (DOCX) [file pone.0248294.s002.docx]

## S2 Fig. Abundance read variability.

To test repeatability of abundance reads from fecal sample, we ran barcoding for two different sub-samples from the same fecal sample. Although the top 5 genera were detected in both sub-samples (occurrence confirmed), the % reads varied in some cases quite dramatically, also changing the relative ranking of the 5 genera (Fig. S8). These differences may highlight the problem of truly homogenizing plant material consisting of different grasses, forbs and shrubs or different parts of the same plant which have different densities of chloroplasts.


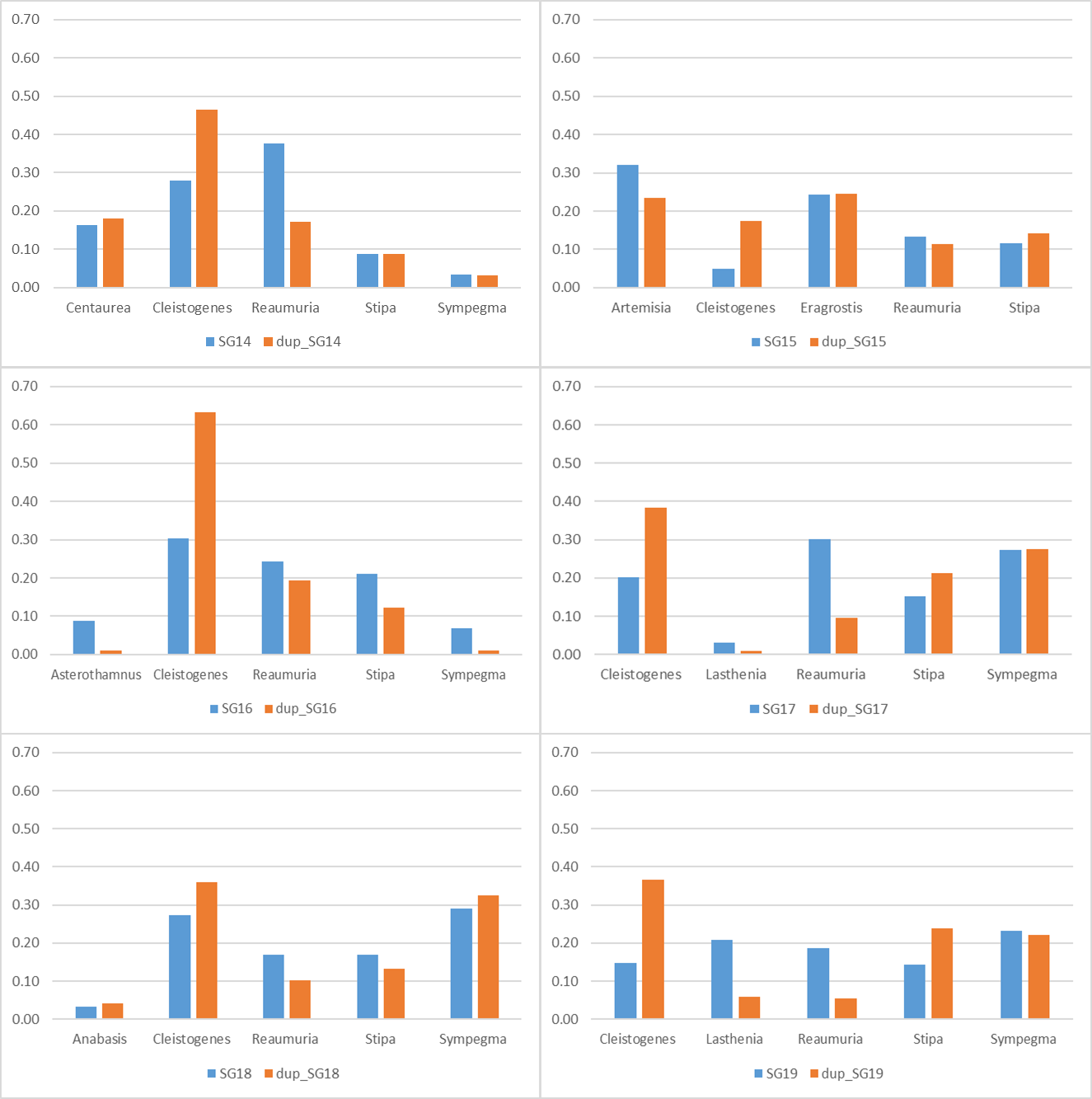


**S2 Fig**. Proportion read abundance for the 5 most common plant genera in the South Gobi Region in the main fecal sample (blue) used for analysis and a second subsample (orange) for six fecal samples from the South Gobi Region to check for variability of read abundance within the same fecal sample.
