# Supplementary material for: Isotope analysis combined with DNA barcoding provide new insights into the dietary niche of khulan in the Mongolian Gobi
Source: PLoS One. 2021 Mar 29;16(3):e0248294. doi: 10.1371/journal.pone.0248294 (PMC8006982; doi:10.1371/journal.pone.0248294)
Supplement: S3 Fig — (DOCX) [file pone.0248294.s003.docx]

## S3 Fig. Core isotopic dietary niche areas.

The core winter isotopic dietary niche widths of hair segments and fecal samples were similar, especially along the carbon axis (SEAB: tail hair = 6.6 ‰^2^, feces = 5.6 ‰^2^ for the South Gobi Region and hair = 8.7 ‰^2^, feces = 9.2 ‰^2^ for the Dzungarian Gobi (Fig. S9)).


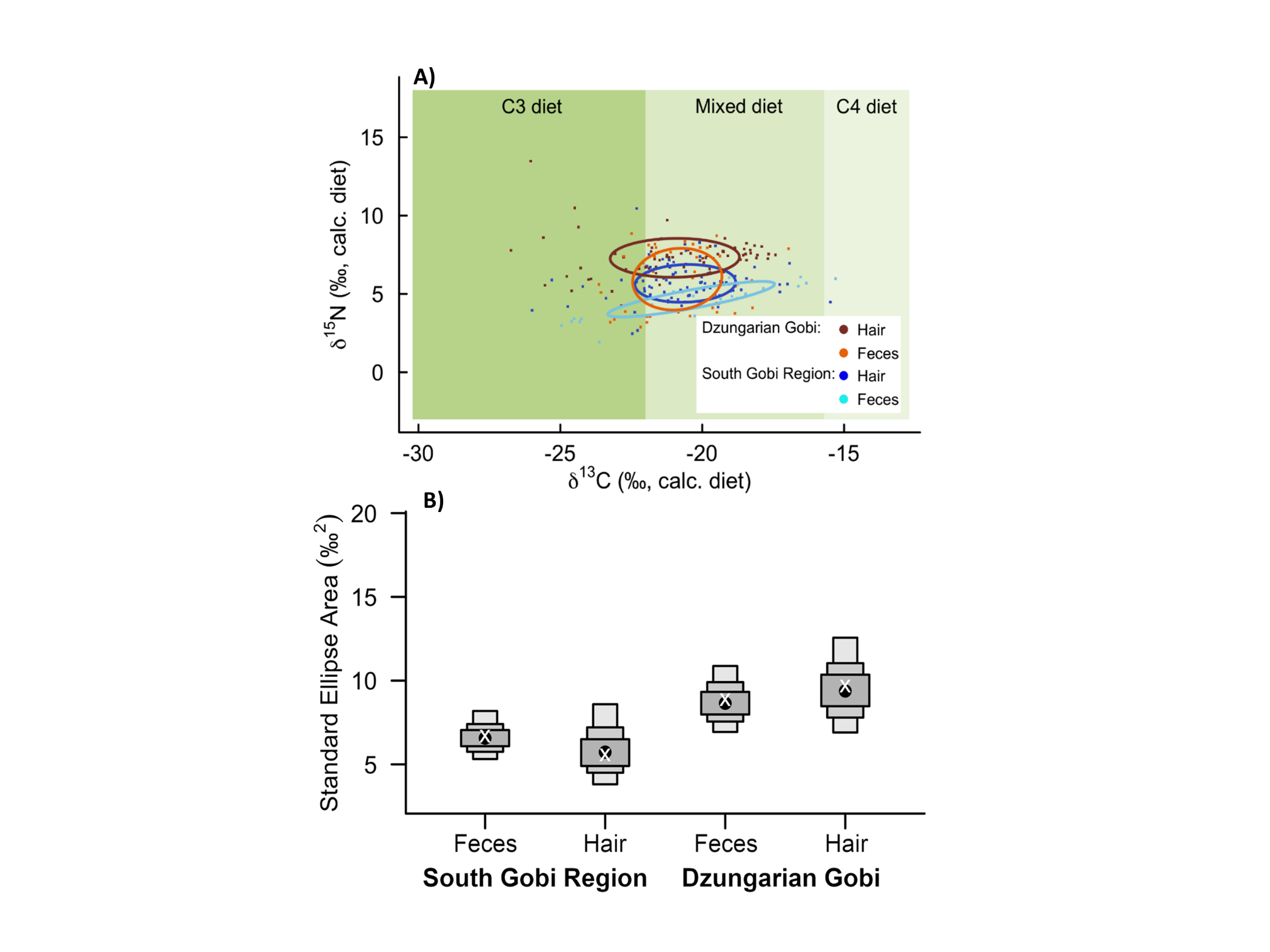


**S3 Fig**. A: Bayesian standard ellipses (SEA_B_) representing estimated core winter isotopic dietary niche widths based on khulan hair and fecal samples, collected in the Dzungarian Gobi and the South Gobi Region. Hair isotope values (winter) for khulan in the Dzungarian Gobi are taken from Burnik Šturm et al. (2017); B: Size of the core isotopic dietary niche areas, expressed as the area of ellipses (‰^2^) in the two-dimensional isotopic space, of khulan´ feces and hair from the SE Gobi and Great Gobi B SPA, respectively. Black dots depict the mode, while the white x represent the true population values. The shaded boxes represent the 50 %, 75 % and 95 % credible intervals from dark to light gray.
