# Supplementary material for: Isotope analysis combined with DNA barcoding provide new insights into the dietary niche of khulan in the Mongolian Gobi
Source: PLoS One. 2021 Mar 29;16(3):e0248294. doi: 10.1371/journal.pone.0248294 (PMC8006982; doi:10.1371/journal.pone.0248294)
Supplement: S4 Fig — (DOCX) [file pone.0248294.s004.docx]

## S4 Fig. Comparison of results with Sugimoto et al. 2018.


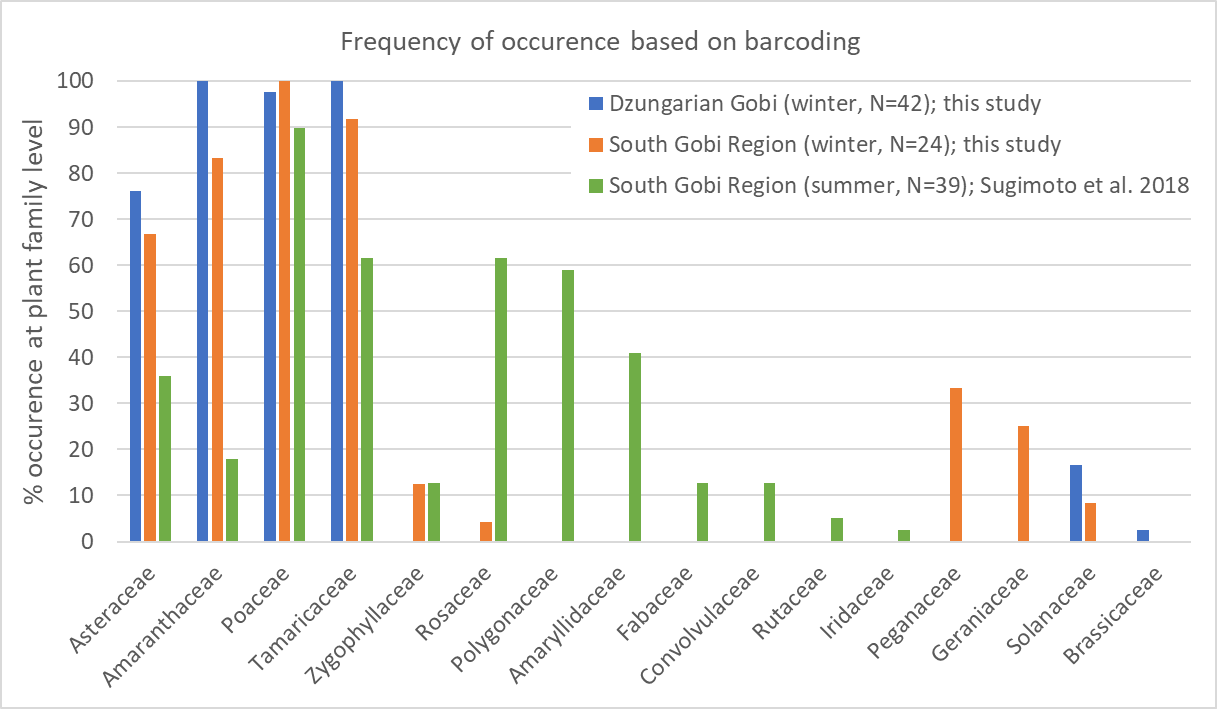


**S4 Fig**. Frequency of occurrence of plant families in two regions of the Mongolian Gobi. For comparison with barcoding results by Sugimoto et al. 2018 see discussion.
