# Supplementary material for: Isotope analysis combined with DNA barcoding provide new insights into the dietary niche of khulan in the Mongolian Gobi
Source: PLoS One. 2021 Mar 29;16(3):e0248294. doi: 10.1371/journal.pone.0248294 (PMC8006982; doi:10.1371/journal.pone.0248294)
Supplement: S1 Table — (DOCX) [file pone.0248294.s005.docx]

## S1 Table. Main habitats and most common plants.

**S1 Table**. Main habitats and most common plants found in the study areas based on the vegetation classification of plant communities in the southern Gobi based on von Wehrden et al. (2009; column Table and No refer to the tables and community numbers within the tables in the publication).

| **Habitat** | **Most common species (>60%; equivalent to IV-V) according to von Wehrden et al. (2009)** | **Table** | **No** |
| --- | --- | --- | --- |
| *Haloxylon ammodendrum* shrub vegetation | [*Haloxylon ammodendron*](https://floragreif.uni-greifswald.de/taxon/?flora_search=Taxon&taxon_id=246) (Ama)** | 6 | 1 |
| Dry desert steppes | [*Stipa glareosa*](https://floragreif.uni-greifswald.de/taxon/?flora_search=Taxon&taxon_id=467) (Poa)*,* [*Anabasis brevifolia*](https://floragreif.uni-greifswald.de/taxon/?flora_search=Taxon&taxon_id=225) (Ama) | 5 | 2 |
| Dry desert steppes | *Stipa glareosa, Anabasis brevifolia,* [*Reaumuria soongarica*](https://floragreif.uni-greifswald.de/taxon/?flora_search=Taxon&taxon_id=587) (Tam) | 5 | 3 |
| Desert steppes of pediments and moister semi-deserts | *Stipa glareosa,* [*Allium mongolicum*](https://floragreif.uni-greifswald.de/taxon/?flora_search=Taxon&taxon_id=15) *(All),* [*Caragana leucophloea*](https://floragreif.uni-greifswald.de/taxon/?flora_search=Taxon&taxon_id=336) (Fab)*,* [*Corispermum mongolicum*](https://floragreif.uni-greifswald.de/taxon/?flora_search=Taxon&taxon_id=244) (Ama)*,* [*Lappula stricta*](https://floragreif.uni-greifswald.de/taxon/?flora_search=Taxon&taxon_id=153) (Bor)*,* [*Iris tenuifolia*](https://floragreif.uni-greifswald.de/taxon/?flora_search=Taxon&taxon_id=365) (Iri)*,* [*Artemisia sublessingiana*](https://floragreif.uni-greifswald.de/taxon/?flora_search=Taxon&taxon_id=1323) (Ast)*,* [*Salsola paulsenii*](https://floragreif.uni-greifswald.de/taxon/?flora_search=Taxon&taxon_id=1251) (Ama) | 3 | 5 |
| Dry desert steppes | [*Stipa gobica*](https://floragreif.uni-greifswald.de/taxon/?flora_search=Taxon&taxon_id=469) (Poa)*,* [*Ajania fruticulosa*](https://floragreif.uni-greifswald.de/taxon/?flora_search=Taxon&taxon_id=43) (Ast)*,* [*Dontostemon senilis*](https://floragreif.uni-greifswald.de/taxon/?flora_search=Taxon&taxon_id=170) (Bra)*, Anabasis brevifolia* | 5 | 1 |
| Dry desert steppes | *Allium mongolicum, Ajania fruticulosa, Stipa glareosa, Nanophyton erinaceum* (Ama), Artemisia sublessingiana* | 5 | 7 |
| Dry scrub vegetation | [*Nitraria sibirica*](https://floragreif.uni-greifswald.de/taxon/?flora_search=Taxon&taxon_id=395) (Nit) | 7 | 9 |
| Mountain steppe | *Stipa gobica,* [*Agropyron cristatum*](https://floragreif.uni-greifswald.de/taxon/?flora_search=Taxon&taxon_id=416) (Poa) | 2 | 10 |
| *Achnatherum splendens* communities on disturbed sites**** | [*Achnatherum splendens*](https://floragreif.uni-greifswald.de/taxon/?flora_search=Taxon&taxon_id=415) (Poa) | 4 | NA |
| *Phragmites australis* community | [*Phragmites australis*](https://floragreif.uni-greifswald.de/taxon/?flora_search=Taxon&taxon_id=452) (Poa) | 8 | NA |
| *Blysmetum rufi* community | [*Triglochin maritima*](https://floragreif.uni-greifswald.de/taxon/?flora_search=Taxon&taxon_id=371) (Jun)*,* [*Halerpestes salsuginosa*](https://floragreif.uni-greifswald.de/taxon/?flora_search=Taxon&taxon_id=516) (Ran)*,* [*Glaux maritima*](https://floragreif.uni-greifswald.de/taxon/?flora_search=Taxon&taxon_id=3031) (Pri) | 8 | 7 |
| Desert steppes of pediments and moister semi-deserts | *Stipa glareosa* | 3 | 2 |
| Desert steppes of pediments and moister semi-deserts | *Stipa glareosa,* [*Krascheninnikovia ceratoides*](https://floragreif.uni-greifswald.de/taxon/?flora_search=Taxon&taxon_id=253) *(synonum: Eurotia ceratoides), Caragana leucophloea* | 3 | 3 |
| Desert steppes of pediments and moister semi-deserts | *Stipa glareosa, Ajania fruticulosa, Caragana leucophloea, Anabasis brevifolia* | 3 | 6 |
| Dry shrub vegetation | [*Ephedra przewalskii*](https://floragreif.uni-greifswald.de/taxon/?flora_search=Taxon&taxon_id=307) (Eph) | 7 | 1 |
| Dry shrub vegetation | *Reaumuria soongorica,* [*Salsola passerina*](https://floragreif.uni-greifswald.de/taxon/?flora_search=Taxon&taxon_id=1250) (Ama)*,*  [*Nitraria sphaerocarpa*](https://floragreif.uni-greifswald.de/taxon/?flora_search=Taxon&taxon_id=396) (Nit) | 7 | 6 |
| Dry shrub vegetation | *Reaumuria soongorica, Salsola passerina,* [*Kalidium gracile*](https://floragreif.uni-greifswald.de/taxon/?flora_search=Taxon&taxon_id=1239) (Ama) | 7 | 8 |
| *Tamarix ramosissima* community | [*Tamarix ramosissima*](https://floragreif.uni-greifswald.de/taxon/?flora_search=Taxon&taxon_id=591) (Tam) | 8 | 4 |

*Not in FloraGREIF 2014; but see page 13 in Tungalag and Boldgiv 2016; **This community was described for the Dzungarian Gobi in von Wehrden et al. 2006, but not specifically delineated in von Wehrden et al. 2009.

**Ama = Amaranthaceae, Poa = Poaceae, Ast = Asteraceae, Tam = Tamaricaceae, All = Alliaceae, Fab = Fabaceae, Bor = Boraginaceae, Iri = Iridaceae, Bra = Brassicaceae, Nit = Nitrariaceae, Jun = Juncaginaceae, Ran = Ranunculaceae, Pri = Primulaceae, Eph = Ephereraceae

**References**

FloraGREIF. 2014. Virtual Flora of Mongolia. <http://floragreif.uni-greifswald.de/floragreif/> at the Computer Centre of University of Greifswald, D-17487 Greifswald, Germany.

Tungalag, R., and B. Boldgiv. 2016. The Flowers of the Mongolian Gobi Desert. Admon Publishing

von Wehrden, H., K. Wesche, and R. Tungalag. 2006. Plant communities of the Great Gobi B Strictly Protected Area, Mongolia. Mongolian Journal of Biological Sciences **4**:63-66.

von Wehrden, H., K. Wesche, and G. Miehe. 2009. Plant communities of the southern Mongolian Gobi. Phytocoenologia **39**:331–376.
