# Supplementary material for: Isotope analysis combined with DNA barcoding provide new insights into the dietary niche of khulan in the Mongolian Gobi
Source: PLoS One. 2021 Mar 29;16(3):e0248294. doi: 10.1371/journal.pone.0248294 (PMC8006982; doi:10.1371/journal.pone.0248294)
Supplement: S2 Table — (DOCX) [file pone.0248294.s006.docx]

## S2 Table. Khulan tail hair samples.

**S2 Table**. Khulan tail hair samples for isotope analysis. Results from tail hair analysis from khulan from the Dzungarian Gobi were already presented in a different context in (Burnik Šturm et al. 2017).

| **Collected** | **Sample** | **Khulan ID** | **Sex** | **Age** | **Tail length (cm)** | **XCO** | **YCO** |
| --- | --- | --- | --- | --- | --- | --- | --- |
| ***Dzungarian Gobi*** | |  |  |  |  |  |  |
| 07/2009 | tail hair | 1 | female | 8 | 51 | 93.44600 | 45.24130 |
| 07/2009 | tail hair | 2 | female | 15 | 41 | 93.54960 | 45.35880 |
| 07/2009 | tail hair | 5 | female | 8-9 | 42 | 93.40840 | 45.22710 |
| 07/2009 | tail hair | 4 | male | 3 | 47 | 93.24040 | 45.42290 |
| 07/2009 | tail hair | 6447 | male | 7 | 53 | 93.39860 | 45.25410 |
| 07/2009 | tail hair | 6441 | male | 15 | 41 | 93.46480 | 45.34110 |
| ***South Gobi Region*** | |  |  |  |  |  |  |
| 08/2013 | tail hair | 13745 | female | 9 | 47 | 106.86977 | 42.90927 |
| 08/2013 | tail hair | 13557 | female | 4 | 47 | 109.09394 | 43.29222 |
| 08/2013 | tail hair | 34408 | female | 6+ | 46 | 106.78283 | 42.93926 |
| 08/2013 | tail hair | 34411 | female | 7 | 45 | 109.17558 | 43.34399 |
| 08/2013 | tail hair | 13747 | male | 7 | 50 | 109.20031 | 43.27670 |
| 08/2013 | tail hair | 13742 | male | 5 | 42 | 107.12234 | 42.80311 |
| 08/2013 | tail hair | 13743 | male | 11 | 44 | 109.19852 | 43.46243 |
| 08/2013 | tail hair | 34407 | male | 10 | 55 | 106.98048 | 42.83515 |

**References**

Burnik Šturm, M., O. Ganbaatar, C. C. Voigt, and P. Kaczensky. 2017. Sequential stable isotope analysis reveals differences in multi-year dietary history of three sympatric equid species in SW Mongolia. J Appl Ecol **54**:1110-1119.
