# Supplementary material for: Isotope analysis combined with DNA barcoding provide new insights into the dietary niche of khulan in the Mongolian Gobi
Source: PLoS One. 2021 Mar 29;16(3):e0248294. doi: 10.1371/journal.pone.0248294 (PMC8006982; doi:10.1371/journal.pone.0248294)
Supplement: S3 Table — (DOCX) [file pone.0248294.s007.docx]

## S3 Table. Khulan fecal samples.

**S3 Table**. Khulan fecal samples for parallel isotope and barcoding analysis.

| **Collected** | **Sample** | **Sample ID** | **Sample age** | **Evidence of khulan origin** | **XCO** | **YCO** |
| --- | --- | --- | --- | --- | --- | --- |
| ***Dzungarian Gobi*** | |  |  |  |  |  |
| 02/2016 | Fecal | 1-20 | fresh | ~350 khulan seen | 93.28410 | 45.30059 |
| 02/2016 | Fecal | 21-25 | fresh | ~350 khulan seen | 93.27808 | 45.30190 |
| 02/2016 | Fecal | 26-30* | 2 weeks old | khulan tracks in the snow | 92.79218 | 45.29841 |
| 02/2016 | Fecal | 31-34 | fresh | 6 khulan seen | 92.53565 | 45.43524 |
| 02/2016 | Fecal | 35* | 1 week old | khulan tracks in the snow | 92.93073 | 45.39009 |
| 02/2016 | Fecal | 36-42 | fresh | 14 khulan seen | 93.01470 | 45.41032 |
| ***South Gobi Region*** | |  |  |  |  |  |
| 02/2016 | Fecal | 1-2** | fresh | 4 khulan seen | 106.9857 | 42.53024 |
| 02/2016 | Fecal | 3-8 | fresh | 27 khulan seen | 107.1155 | 42.57395 |
| 02/2016 | Fecal | 9-13 | fresh | 35 khulan seen | 107.1077 | 42.82387 |
| 02/2016 | Fecal | 14 | fresh | 5 khulan seen | 108.8637 | 42.47329 |
| 02/2016 | Fecal | 15-16 | fresh | 44 khulan seen | 107.8867 | 42.50294 |
| 02/2016 | Fecal | 17-19 | fresh | 17 khulan seen | 108.8982 | 42.87511 |
| 02/2016 | Fecal | 20-22 | fresh | 8 khulan seen | 108.4942 | 43.41512 |
| 02/2016 | Fecal | 23-25 | fresh | 9 khulan seen | 108.4092 | 43.51966 |

*For these six older fecal samples, collected in an area were only khulan tracks were seen in the snow, we confirmed the species origin of these by amplifying a 193 basepair section of mtDNA control region DNA using primers designed from published sequences as per (Kaczensky et al. 2018). Also see Appendix S5.

**sample #2 was lost for barcoding analysis resulting in a sample size of N=25 for the isotope results and N=24 for the barcoding results.

**References**

Kaczensky, P., E. Kovtun, R. Habibrakhmanov, M.-R. Hemami, A. Khaleghi, J. D. C. Linnell, E. Rustamov, S. Sklyarenko, C. Walzer, S. Zuther, and R. Kuehn. 2018. First population-level genetic analysis of free-ranging Asiatic wild ass populations in Central Asia - implications for conservation. Conservation Genetics **19**:1169–1184.
