# Supplementary material for: Isotope analysis combined with DNA barcoding provide new insights into the dietary niche of khulan in the Mongolian Gobi
Source: PLoS One. 2021 Mar 29;16(3):e0248294. doi: 10.1371/journal.pone.0248294 (PMC8006982; doi:10.1371/journal.pone.0248294)
Supplement: S4 Table — (DOCX) [file pone.0248294.s008.docx]

## S4 Table. Primers for DNA barcoding.

**S4 Table**. Primer sequences used to amplify equid mtDNA and plant dietary DNA from fecal samples of khulan.

| Primer Name | Sequence | Source Genome |
| --- | --- | --- |
| Eprz_Dloop_2_F | GTCAGTATCAGATTATACCCCC | mtDNA (Equid; Kaczensky et et al. 2018) |
| Eprz_Dloop_2_R | TGGAACATGGGTTGTGATATG | mtDNA (Equid; Kaczensky et et al. 2018) |
| trnL(UAA)g | TCG TCG GCA GCG TCA GAT GTG TAT AAG AGA CAG GGGCAATCCTGAGCCAA | Chloroplast (Plant; Kartzinel et al. 2015) |
| trnL(UAA)h | GTC TCG TGG GCT CGG AGA TGT GTA TAA GAG ACA G CCATTGAGTCTCTGCACCTATC | Chloroplast (Plant) |
| ITS1-F | TCG TCG GCA GCG TCA GAT GTG TAT AAG AGA CAG GATATCCGTTGCCGAGAGTC | Nuclear (Plant; Kartzinel et al. 2015)  all plant orders |
| ITS1Ast-R | GTC TCG TGG GCT CGG AGA TGT GTA TAA GAG ACA G CGGCACGGCATGTGCCAAGG | Nuclear (Plant)  family Asteraceae |
| ITS1Poa-R | GTC TCG TGG GCT CGG AGA TGT GTA TAA GAG ACA G CCGAAGGCGTCAAGGAACAC | Nuclear (Plant; Kartzinel et al. 2015)  family Poaceae |

**Reads obtained with trnL only and the full primer set**

The trnL primer alone produced almost 39 million reads, whereas the addition of the two family specific primers (ITS1-Ast and ITS1-Poa) accounted for a further 68 million reads. With trnL alone, we retrieved 69 genera in total and just seven after filtering for low abundance. The full primer set identified 169 genera from the raw data and 39 after filtering. Most of the missing genera belonged to the families Asteraceae (9), Poaceae (9), Amaranthaceae (11), and the remaining 5 to one family represented by a single genus each.

**References**

Kaczensky, P., E. Kovtun, R. Habibrakhmanov, M.-R. Hemami, A. Khaleghi, J. D. C. Linnell, E. Rustamov, S. Sklyarenko, C. Walzer, S. Zuther, and R. Kuehn. 2018. First population-level genetic analysis of free-ranging Asiatic wild ass populations in Central Asia - implications for conservation. Conservation Genetics **19**:1169–1184.

Kartzinel, T. R., P. A. Chen, T. C. Coverdale, D. L. Erickson, W. J. Kress, M. L. Kuzmina, D. I. Rubenstein, W. Wang, and R. M. Pringle. 2015. DNA metabarcoding illuminates dietary niche partitioning by African large herbivores. PNAS **112**:7873-8154.
