# Supplementary material for: Isotope analysis combined with DNA barcoding provide new insights into the dietary niche of khulan in the Mongolian Gobi
Source: PLoS One. 2021 Mar 29;16(3):e0248294. doi: 10.1371/journal.pone.0248294 (PMC8006982; doi:10.1371/journal.pone.0248294)
Supplement: S5 Table — (DOCX) [file pone.0248294.s009.docx]

## S5 Table. Plant genera identified with DNA barcoding.

**S5 Table**. Characteristics, distribution and number of 23 plant genera detected with barcoding (>0.5% of total reads or >0.5% reads in individual scats) in 66 khulan winter feces collected in the Dzungarian Gobi and South Gobi Region in winter 2016.

Hyperlinks lead to discriptions and distribution in (FloraGREIF 2014). C3/C4 characterisation is based on Pyankov et al. 200 and Wang 2007. ? = 2 genera not recorded for the Gobi, ?? = 1 genus not recorded for Mongolia/Asia.

| **Genus** | **C3/C4** | **Genus occurs in**  (found in scats) | | | **N species**  **in MON** | **Characteristics as forage plants**  **(Jigjidsuren and Johnson 2003)** |
| --- | --- | --- | --- | --- | --- | --- |
|  |  | **Mongolia**  **(MON)** | **DG** | **SGR** |  |  |
| **Asteraceae** – 9 genera detected in khulan feces | | | | | | |
| [*Artemisia*](https://floragreif.uni-greifswald.de/taxon/?flora_search=Taxon&gen_id=62) | C3 | yes | yes (yes) | yes (yes) | 98 | Some species very nutritious for sheep, goats, camels in summer and very palatable for horses and cattle in spring & winter. |
| [*Asterothamnus*](https://floragreif.uni-greifswald.de/taxon/?flora_search=Taxon&gen_id=64) | C3 | yes | yes  (no) | yes  (yes) | 5 | NA |
| [*Centaurea*](https://floragreif.uni-greifswald.de/taxon/?flora_search=Taxon&gen_id=71)*?* | C3 | yes | **no**  (yes) | **no**  (yes) | 1 | NA, this genus should only occur in the NW mountains but may have a closely related species in the lowlands |
| *Lasthenia??* | C3 | no | no  (yes) | no  (yes) | NA | N-American species, potentially there is a closely related species in the Gobi |
| [*Saussurea*](https://floragreif.uni-greifswald.de/taxon/?flora_search=Taxon&gen_id=114) | C3 | yes | yes  (yes) | yes  (no) | 49 |  |
| [*Rhaponticum*](https://floragreif.uni-greifswald.de/taxon/?flora_search=Taxon&gen_id=112)*?* | C3 | yes | no  (yes) | no  (yes) | 1 | NA, this genus should only occur in the mountains but may have a closely related species in the lowlands |
| [*Lactuca*](https://floragreif.uni-greifswald.de/taxon/?flora_search=Taxon&gen_id=96) | C3 | yes | yes (yes) | yes (yes) | 2 | NA |
| [*Aster*](https://floragreif.uni-greifswald.de/taxon/?flora_search=Taxon&gen_id=63) | C3 | yes | yes  (no) | yes  (yes) | 4 | Good nutritional value, but poorly palatable to horses |
| [*Ancathia*](https://floragreif.uni-greifswald.de/taxon/?flora_search=Taxon&gen_id=58) | C3 | yes | yes  (yes) | no  (no) | 1 | NA |
| **Amaranthaceae (formerly Chenopodiaceae)** – 12 genera detected in khulan feces | | | | | | |
| [*Haloxylon*](https://floragreif.uni-greifswald.de/taxon/?flora_search=Taxon&gen_id=271) | C4 | yes | yes (yes) | yes (yes) | 1 | Camels graze young shoots year round, sheep & goats select fallen buds in winter. |
| [*Anabasis*](https://floragreif.uni-greifswald.de/taxon/?flora_search=Taxon&gen_id=260) | C4 | yes | yes (yes) | yes (yes) | 7 | All poisonous except *A. brevifolia*, camels graze it except summer, in summer it causes diarrhoea, livestock feeding on it fattens well. |
| [*Salsola*](https://floragreif.uni-greifswald.de/taxon/?flora_search=Taxon&gen_id=281) | C4 | yes | yes (yes) | yes (yes) | 13 | High nutrient values throughout year, primary feed except in summer, large animals prefer to feed on it in winter & spring. |
| [*Kochia*](https://floragreif.uni-greifswald.de/taxon/?flora_search=Taxon&gen_id=274) | C4 | yes | yes (yes) | yes (yes) | 6 | High forage value, prime forage species in autumn-winter. |
| [*Agriophyllum*](https://floragreif.uni-greifswald.de/taxon/?flora_search=Taxon&gen_id=259) | C3 | yes | no  (no) | yes  (yes) | 1 | NA |
| [*Krascheninnikovia (Eurotia)*](https://floragreif.uni-greifswald.de/taxon/?flora_search=Taxon&gen_id=275) | C3 | yes | yes (yes) | yes  (no) | 1 | “Winterfat”. High nutrient forage year round, dried forage stores well. |
| [*Sympegma*](https://floragreif.uni-greifswald.de/taxon/?flora_search=Taxon&gen_id=283) | C3 | yes | yes (yes) | yes (yes) | 1 | NA |
| [*Nitraria*](https://floragreif.uni-greifswald.de/taxon/?flora_search=Taxon&gen_id=428) | C4 | yes | yes  (yes) | yes  (yes) | 3 | Horses and cattle do not graze, sheep and goats infrequently. Moderately palatable to camels in summer and autumn, poor palatability in winter. |
| [*Agriophyllum*](https://floragreif.uni-greifswald.de/taxon/?flora_search=Taxon&gen_id=259) | C3 | yes | yes  (no) | yes  (yes) | 1 | NA |
| [*Corispermum*](https://floragreif.uni-greifswald.de/taxon/?flora_search=Taxon&gen_id=268) | C3 | yes | yes  (no) | yes  (yes) | 7 | NA |
| [*Chenopodium*](https://floragreif.uni-greifswald.de/taxon/?flora_search=Taxon&gen_id=266) | C4 | yes | yes  (no) | yes  (yes) | 17 | Nutrient value considered average. Some species not grazed when dry, others could be used as dry hay and silage. |
| [*Halogeton*](https://floragreif.uni-greifswald.de/taxon/?flora_search=Taxon&gen_id=270)*?* | C4 | yes | yes  (no) | no  (yes) | 1 | Should not be found so far east but may have a closely related species in the lowlands. |
| **Poaceae** – 10 genera detected in khulan feces | | | | | | |
| [*Stipa*](https://floragreif.uni-greifswald.de/taxon/?flora_search=Taxon&gen_id=526) | C3 | yes | yes (yes) | yes (yes) | 19 | Palatable to all livestock throughout the year. |
| [*Psammochloa*](https://floragreif.uni-greifswald.de/taxon/?flora_search=Taxon&gen_id=517) | C3 | yes | no  (no) | yes  (yes) | 1 | NA |
| [*Eragrostis*](https://floragreif.uni-greifswald.de/taxon/?flora_search=Taxon&gen_id=496) | C4 | yes | yes (yes) | yes (yes) | 4 | Highly palatable to cattle and horses in summer, poor palatability in winter. |
| [*Cleistogenes*](https://floragreif.uni-greifswald.de/taxon/?flora_search=Taxon&gen_id=489) | C4 | yes | yes (yes) | yes (yes) | 5 | Palatable to all livestock throughout the year. |
| [*Aristida*](https://floragreif.uni-greifswald.de/taxon/?flora_search=Taxon&gen_id=479) | C4 | yes | no  (no) | yes  (yes) | 1 | Palatable to camels and small livestock throughout the year. |
| [*Elymus*](https://floragreif.uni-greifswald.de/taxon/?flora_search=Taxon&gen_id=493) */ Lymus* | C3 | yes | yes  (yes) | es  (no) | 16 | Mostly high palatability for large livestock with moderate to high nutrient values. |
| [*Ptilagrostis*](https://floragreif.uni-greifswald.de/taxon/?flora_search=Taxon&gen_id=519) | C3 | yes | yes  (yes) | yes  (no) | 3 | Palatable to horses and cattle, highly nutritious. |
| [*Phragmites*](https://floragreif.uni-greifswald.de/taxon/?flora_search=Taxon&gen_id=513) | C3 | yes | yes  (yes) | yes  (no) | 1 | Highly palatable in spring and summer, moderate nutritional value. Animals survive well on it in winter. |
| [*Psathyrostachys*](https://floragreif.uni-greifswald.de/taxon/?flora_search=Taxon&gen_id=518) | C3 | yes | yes  (yes) | yes  (no) | 2 | “Wild rye”. Good nutritional value, drought and cold resistance. |
| [*Tragus*](https://floragreif.uni-greifswald.de/taxon/?flora_search=Taxon&gen_id=527) | C4 | yes | no  (no) | yes  (yes) | 1 | NA |
| **Tamaricaceae** | | | | | | |
| [*Reaumuria*](https://floragreif.uni-greifswald.de/taxon/?flora_search=Taxon&gen_id=649) | C3 | yes | yes (yes) | yes (yes) | 1 | Camels feed on it in spring, all livestock moderately graze it in winter |
| **Geraniaceae** | | | | | | |
| [*Erodium*](https://floragreif.uni-greifswald.de/taxon/?flora_search=Taxon&gen_id=365) | C3 | yes | yes  (no) | yes  (yes) | 3 | NA |
| **Peganaceae** | | | | | | |
| [*Peganum*](https://floragreif.uni-greifswald.de/taxon/?flora_search=Taxon&gen_id=461) | C3 | yes | yes  (no) | yes  (yes) | 2 | Often around camps, given in small quantities when dry to young camels and weakened animals during winter in desert areas with food shortage, considered poisonous. |
| **Zygophyllaceae** | | | | | | |
| [[*Tribulus*](https://floragreif.uni-greifswald.de/taxon/?flora_search=Taxon&gen_id=665)](https://floragreif.uni-greifswald.de/taxon/?flora_search=Taxon&gen_id=365) | C4 | yes | yes  (no) | yes  (yes) | 1 | NA |
| **Solanaceae** | | | | | | |
| [*Solanum*](https://floragreif.uni-greifswald.de/taxon/?flora_search=Taxon&gen_id=646) | C3 | yes | yes  (yes) | yes  (yes) | 2 | NA |
| **Convolvulaceae** | | | | | | |
| [*Convolvulus*](https://floragreif.uni-greifswald.de/taxon/?flora_search=Taxon&gen_id=288) | C3 | yes | yes  (no) | yes  (yes) | 5 | Good summer-autumn grazing to fatten animals |
| **Brassicaceae** | | | | | | |
| [*Lepidium*](https://floragreif.uni-greifswald.de/taxon/?flora_search=Taxon&gen_id=195) | C3 | yes | yes  (yes) | yes  (no) | 10 | NA |
| **Rosaceae** | | | | | | |
| [*Prunus*](https://floragreif.uni-greifswald.de/taxon/?flora_search=Taxon&gen_id=605) | **C3** | yes | Yes  (no) | Yes  (yes) | 1 | NA |

**References**

Jigjidsuren, S., and D. A. Johnson. 2003. Forage Plants in Mongolia. Admon Press, Ulaanbaatar, Mongolia. [in Mongolian and English]

FloraGREIF. 2014. Virtual Flora of Mongolia. <http://floragreif.uni-greifswald.de/floragreif/> at the Computer Centre of University of Greifswald, D-17487 Greifswald, Germany.

Pyankov, V. I., P. D. Gunin, S. Tsoog, and C. C. Black. 2000. C4 plants in the vegetation of Mongolia: their natural occurrence and geographical distribution in relation to climate. Oecologia **123**:15-31.

Wang, R. Z. 2007. C4 plants in the deserts of China: occurrence of C4 photosynthesis and its morphological functional types. Photosynthetica **45**:167-171.
