# Supplementary material for: Isotope analysis combined with DNA barcoding provide new insights into the dietary niche of khulan in the Mongolian Gobi
Source: PLoS One. 2021 Mar 29;16(3):e0248294. doi: 10.1371/journal.pone.0248294 (PMC8006982; doi:10.1371/journal.pone.0248294)
Supplement: S6 Table — (DOCX) [file pone.0248294.s010.docx]

## S6 Table. Comparison of results with Xu et al. 2018.

**S6 Table**. Comparison of winter diet in the Dzungarian Gobi based on micro-histological analysis of 25 fecal samples from Kalamaili Mountain Ungulate Nature Reserve in Northern Xinjiang, China (rearranged after Xu et al. 2018) and based on barcoding of 42 fecal samples from Great Gobi B Strictly Protected Area in adjacent Mongolia (this study).

Additional genera found in our study were aggregated and added to the original list by Xu et al. 2012 under the family with the extension “- others (our study)”.

Grey cells – functional groups.

Yellow rows– newly detected diet items at genus level with barcoding (this study).

Orange rows– large mismatch in the importance of the diet item between results by Xia et al. 2012 with both diet calculation methods based on barcoding in this study.

s=shrubs, f = forbs (based on Xu et al. 2012, FloraGREIF 2014, and Tungalag and Boldgiv 2016)

| **Plant family (growth form)** | **Plant species / genera** | **Diet composition of khulan winter scats in the Dzungarian Gobi** | | |
| --- | --- | --- | --- | --- |
|  |  | **Xu et al. 2012, NW China Micro-histology (N=25) [relative density]** | **this study Barcoding (N=42) [realative RA]** | **this study Barcoding (N=42) [realative FOO]** |
| **Graminoids** |  | **14.8** | **23.5** | **26.0** |
| Poaceae | *Stipa glareosa* | 11.2 | 21.6 | 15.6 |
| Poaceae | *Stipa glareosa* |  |  |  |
| Poaceae | *Achnatherum splendens* | 2.2 |  |  |
| Poaceae | *Phragmites australis* | 1.4 |  | 0.8 |
| Poaceae | *Eragostis* |  | 1.2 | 3.1 |
| Poaceae | *Leymus /Elymus* |  | 0.7 | 1.9 |
| Poaceae | *Cleistogenes* |  |  | 1.9 |
| Poaceae | *Poa* |  |  | 1.5 |
| Poaceae | *Ptilagrostis* |  |  | 0.8 |
| Poaceae | *Psathyrostachys* |  |  | 0.4 |
| **Shrubs (s) / forbs (f)** |  | **85.2** | **76.5** | **74.0** |
| Amaranthaceae (s) | *Haloxylon ammodendron* | 16.5 | 17.3 | 16.0 |
| Amaranthaceae (s) | *Krascheninnikovia ceratoides / Ceratoides latens* | 5.2 | 0.8 | 3.8 |
| Amaranthaceae (s) | *Anabasis sp.* | 28.6 | 0.6 | 4.6 |
| Amaranthaceae (f) | *Salsola affinis* | 4.8 | 1.2 | 3.8 |
| Amaranthaceae (f) | *Salsola subcrassa* | 6 |  |  |
| Amaranthaceae (f or s) | *Other* |  |  |  |
| Amaranthaceae (f) | *Ceratocarpus arenarius* | 1.6 |  |  |
| Amaranthaceae (s) | *Sympegma* |  |  | 1.9 |
| Amaranthaceae (s) | *Nitraria* |  |  | 0.8 |
| Amaranthaceae (f or s) | *Kochia* |  |  | 0.8 |
| Amaranthaceae (s) | *Kalidium* |  |  | 0.4 |
| Tamaricaceae (s) | *Tamarix sp.* | 0.4 |  |  |
| Tamaricaceae (s) | *Reaumuria soongorica* | 17.3 | 41.8 | 16.0 |
| Asteraceae (s) | *Seriphidium santolinum / Artemisia santolina* | 4.6 | 8.6 | 12.2 |
| Asteraceae (s or f) | *Artemesia* |  |  |  |
| Asteraceae (f) | *Lactuca* |  |  | 1.5 |
| Asteraceae (f) | *Saussurea* |  |  | 0.8 |
| Asteraceae (f) | *Ancathia* |  |  | 0.4 |
| Asteraceae | *Centaurea?* |  | 2.2 | 2.7 |
| Asteraceae | *Rhaponticum?* |  |  | 1.9 |
| Asteraceae | *Lasthenia??* |  |  | 3.4 |
| Ephedraceae (s) | *Ephedra przewalskii* | 0.2 |  |  |
| Solanaceae (s) | *Solanum* |  | 4.3 | 2.7 |
| Brassicaceae (f) | *Lepidium* |  |  | 0.4 |
| *Other* (?) |  |  |  |  |

**References**

FloraGREIF. 2014. Virtual Flora of Mongolia. <http://floragreif.uni-greifswald.de/floragreif/> at the Computer Centre of University of Greifswald, D-17487 Greifswald, Germany.

Tungalag, R., and B. Boldgiv. 2016. The Flowers of the Mongolian Gobi Desert. Admon Publishing.

Xu, W., C. Xia, W. Yang, D. A. Blank, J. Qiao, and W. Liu. 2012. Seasonal diet of Khulan (Equidae) in Northern Xinjiang, China. Italian Journal of Zoology **79**:92-99.
