# Supplementary material for: Isotope analysis combined with DNA barcoding provide new insights into the dietary niche of khulan in the Mongolian Gobi
Source: PLoS One. 2021 Mar 29;16(3):e0248294. doi: 10.1371/journal.pone.0248294 (PMC8006982; doi:10.1371/journal.pone.0248294)
Supplement: S1 File — (DOCX) [file pone.0248294.s011.docx]

## S1 File. Stable isotope analysis details.

### Isotope analysis

All isotope analyses were conducted at the stable isotope facility at the Leibniz Institute for Zoo and Wildlife Research (IZW), Berlin.

Hair samples and weighted fecal material were enclosed into tin or silver cups (IVA Analysetechnik e.K., Meerbuch, Germany). Stable carbon and nitrogen isotope ratios were measured using an elemental analyzer (Flash EA, ThermoFisher Scientific, Bremen, Germany) connected in continuous mode to a stable isotope ratio mass spectrometer (Delta V-Advantage, ThermoFisher Scientific, Bremen, Germany). Sample isotope ratios were compared to international standards (V-PDB and air N) and expressed as *δ*^13^C and *δ*^15^N values in part per mill deviation from standard values.

For measuring stable isotope ratios of non-exchangeable hydrogen, samples were dried in a drying oven over 24 hours at 50°C and then placed into silver capsules (IVA Analysetechnik E.K., Meerbusch, Germany). Loaded capsules were then placed in a Zero Blank autosampler (Costech Analytical Technologies Inc. Italy) where they were flushed for at least an hour with chemically pure helium before dropping into the elemental analyzer (HT Elementaranalysator HEKAtech GmbH, Wegberg, Germany). The EA was connected via an interface (Finnigan Conflo III, ThermoFisher Scientific Bremen, Germany) to the stable isotope ratio mass spectrometer (Delta V advantage, ThermoFisher Scientific, Bremen, Germany).

To overcome the problem of uncontrolled hydrogen isotopic exchange between keratin and isotopically variable ambient moisture in the laboratory, we used the comparative equilibration method described by Wassenaar and Hobson (2003). Samples were analyzed together with previously calibrated in-house keratin hydrogen isotope reference materials: SWE-SHE (-167.9±1‰, sheep wool), ESP-SHE (-108.3±1‰, sheep wool), and AFR-GOA (-66.3±0.9‰, goat wool). Details on the preparation of the in-house keratin standards are described in Popa-Lisseanu *et al.* (2012). Stable hydrogen isotope ratios are expressed as delta value in per mille deviation from the international standard V-SMOW. Precision of the measurements was always better than 0.1 ‰ for *δ*^13^C and *δ*^15^N, and 1.0 ‰ for non-exchangeable *δ*^2^H values based on the repeated analysis of the laboratory standards, calibrated with the international standards.

### Plant samples for mixing model

To obtain isotope values for C3 and C4 plants for the dual end-member mixing model from (Codron et al. 2007) we collected 240 plant samples in the Dzungarian Gobi in 2012 and 2013. We focused on plants known to be frequently consumed by wild equids based on literature (Bannikov 1981; Xu et al. 2012) and direct observation by protected area staff and local herders.

C3 plants were represented by 13 different plant species (*Stipa* sp., *Achnatherum splendens*, *Agropyron* sp., *Ajania* sp., *Allium* sp., *Artemisia* sp., *Caragana leucophloea*, *Elymus* sp., *Krascheninnikovia ceratoides (synonum: Eurotia ceratoides)*, *Festuca* sp., *Reaumuria soongorica*, *Zygophyllum* sp., *Phragmites australis*) and C4 species by 2 different plant species (*Haloxylon ammondendron* and *Anabasis brevifolia*), as described in detail in (Burnik Šturm et al. 2017). In the South Gobi Region, we collected 8 samples of *Stipa* sp. and 4 of *Haloxylon ammondendron* in 2013 for comparison of their stable isotope composition with previously obtained data from the Dzungarian Gobi.

Each plant sample comprised small parts from 5-10 plants of the same species. We collected the samples in labelled paper envelopes and dried them in the field. In the laboratory, we re-dried the plant samples, homogenized them by grinding, and weighed (2.0-2.5 mg) them into tin cups for C and N stable isotope analysis.

To convert raw hair isotope values (*δ*^13^C_hair_ and *δ*^15^N_hair_) into diet values (*δ*^13^C_diet_ and *δ*^15^N_diet_), we used the dual mixing model of (Cerling et al. 2007). C3 and C4 end–members for the model were based on the average *δ*^13^C values (±1 σ) of the 240 plant samples collected in the Dzungarian Gobi in 2012 and 2013 (i.e. -25.5 ± 1.3 ‰ for C_3_ plants and -13.5 ± 0.5 ‰ for C_4_ plants (Burnik Šturm et al. 2017) and some additional samples from the South Gobi Region in 2016 to confirm they fall within the values from plants collected in the Dzungarian Gobi. The model of (Cerling et al. 2007) takes into account that hair isotope values represent dietary inputs from three pools: the short-term (with 0.5-day half-life and with fraction contributions: 41 % for C and 40 % for N), intermediate (4 days, 15 % for C, 12 % for N) and long-term pool (138 days, 44 % for C, 48 % for N). We used diet–hair fractionation factors for horses on a low protein diet (Ayliffe et al. 2004; Sponheimer et al. 2003): 2.7 ‰ for C and 1.9 ‰ for N.

To calculate the % of C4 diet in the feces, we used a dual end-member mixing model from (Codron et al. 2007), where % C4 = (*δ*^13^C_C3 plants_ + ∆*δ*^13^C - *δ*^13^C_feces_) / (*δ*^13^C_C3 plants_ -∆*δ*^13^C_C4 plants_).To convert raw fecal isotope values (*δ*^13^C_feces_ and *δ*^15^N_feces_) into diet values, we used the following diet-feces fractionation factors: -0.7 ‰ for N and 1 ‰ for C (Codron et al. 2009; Sponheimer et al. 2003; Sutoh et al. 1987).

To visually present the site-specific trends of *δ*^13^C_diet_, and *δ*^2^H_diet_ over time, we used generalized additive models with “individual” as random factor and with integrated smoothness estimation as described in (Burnik Šturm et al. 2015 & 2017). We used histograms and QQ plots to visually inspect residuals for normality and independence. To determine the impact of pasture seasonality on diet, we aligned the diet and isotope profiles with time-matched 16-day Normalized Difference Vegetation Index (NDVI) values recalculated slightly different than in (Burnik Šturm et al. 2017), but in identical ways for both study areas to allow direct comparison (see below).

We used a Bayesian approach to estimate species specific core isotopic dietary niches based on bivariate, ellipse–based metrics (for *δ* ^13^C and *δ* ^15^N values) using SIBER implemented in the R package SIAR (Version 4.2) (Jackson et al. 2011). Fecal and tail hair isotope data (CN) from the South Gobi Region were normally distributed, and the winter tail hair isotope data (N=75) from the Dzungarian Gobi showed a near-normal distribution and we did not apply transformations. Core isotopic niche sizes are expressed as the standard Bayesian ellipse area (SEA_B_) in ‰^2^, defined by a subsample containing 40% of bivariate data (for results see Appendix S9).

### NDVI calculations for seasonal alignment

We downloaded 250m, 16-day composite *MODIS/Terra Vegetation Indices* data (Version 6, MOD13Q1, Didan 2015) from the USGS website (<https://e4ftl01.cr.usgs.gov/MOLT/MOD13Q1.006/>) for the Dzungarian Gobi for the dates 2004-01-01 to 2010-12-19 and for the South Gobi Region for the dates 2010-01-01 to 2018-03-22. We mosaiced and re-projected the tiles (from *Sin* to *Geographic*), extracted two layers: 1) NDVI index, and 2) NASA’s “pixel reliability” layer (Didan 2015), and loaded the layers into PostgreSQL database tables, all using a custom R function and NASA’s *MODIS Reprojection Tool* (<https://lpdaac.usgs.gov/tools/modis_reprojection_tool>).

Images with a significant number of pixels containing snow or cloud can cause biased estimates of average NDVI values. We therefore simplified the quality control layer by converting all categories except “*Good data*” to missing-data values, then used the resulting layer as a mask to exclude questionable pixels from the NDVI data. We clipped the final layer to the convex hull of khulan tracks in each of the two study areas and calculated an arithmetic average NDVI value (ignoring missing values) for each date in the time series.

**References**

Ayliffe, L. K., T. E. Cerling, T. Robinson, A. G. West, M. Sponheimer, B. H. Passey, J. Hammer, B. Roeder, M. D. Dearing, and J. R. Ehleringer. 2004. Turnover of carbon isotopes in tail hair and breath CO2 of horses fed an isotopically varied diet. Oecologia **139**:11-22.

Bannikov, A. G. 1981. The Asian Wild Ass. Lesnaya Promyshlennost, Moscow, Russia. [original in Russian, English translation by M. Proutkina, Zoological Society of San Diego].

Burnik Šturm, M., O. Ganbaatar, C. C. Voigt, and P. Kaczensky. 2017. Sequential stable isotope analysis reveals differences in multi-year dietary history of three sympatric equid species in SW Mongolia. J Appl Ecol **54**:1110-1119.

Cerling, T. E., L. K. Ayliffe, M. D. Dearing, J. R. Ehleringer, B. H. Passey, D. W. Podlesak, A. M. Torregrossa, and A. G. West. 2007. Determining biological tissue turnover using stable isotopes: the reaction progress variable. Oecologia **151**:175-189.

Codron, D., J. A. Lee-Thorp, M. Sponheimer, and J. Codron. 2007. Stable carbon isotope reconstruction of ungulate diet changes through the seasonal cycle. South African Journal of Wildlife Research **37**:117-125.

Didan, K., A. B. Munoz, R. Solano, and A. Huete. 2015. MODIS Vegetation Index User’s Guide (MOD13 Series). Vegetation Index and Phenology Lab, The University of Arizona College of Agriculture and Life Sciences, Tuscon, Arizona, USA. Available from: <https://lpdaac.usgs.gov/documents/103/MOD13_User_Guide_V6.pdf>

Jackson, A. L., R. Inger, A. C. Parnell, and S. Bearhop. 2011. Comparing isotopic niche widths among and within communities: SIBER - Stable Isotope Bayesian Ellipses in R. J Anim Ecol **80**:595-602.

Popa-Lisseanu, A. G., K. Sorgel, A. Luckner, L. I. Wassenaar, C. Ibanez, S. Kramer-Schadt, M. Ciechanowski, T. Gorfol, I. Niermann, G. Beuneux, R. W. Myslajek, J. Juste, J. Fonderflick, D. H. Kelm, and C. C. Voigt. 2012. A triple-isotope approach to predict the breeding origins of European bats. PLoS ONE **7**:e30388.

Sponheimer, M., T. Robinson, L. Ayliffe, B. Roeder, J. Hammer, B. Passey, A. West, T. Cerling, D. Dearing, and J. Ehleringer. 2003. Nitrogen isotopes in mammalian herbivores: hair d^15^N values from a controlled feeding study. International Journal of Osteoarchaeology **13**:80-87.

Sutoh, M., T. Koyama, and T. Yoneyama. 1987. Variations of Natural 15N Abundances in the Tissues and Digesta of Domestic Animals. Radioisotopes **36**:74-77.

Wassenaar, L. I. and K. A. Hobson. 2003. Comparative equilibration and online technique for determination of non-exchangeable hydrogen of keratins for animal migration studies. Isotopes in Environmental and Health Studies, 39, 211–217.

Xu, W., C. Xia, W. Yang, D. A. Blank, J. Qiao, and W. Liu. 2012. Seasonal diet of Khulan (Equidae) in Northern Xinjiang, China. Italian Journal of Zoology **79**:92-99.
