# Supplementary material for: Isotope analysis combined with DNA barcoding provide new insights into the dietary niche of khulan in the Mongolian Gobi
Source: PLoS One. 2021 Mar 29;16(3):e0248294. doi: 10.1371/journal.pone.0248294 (PMC8006982; doi:10.1371/journal.pone.0248294)
Supplement: S2 File — (DOCX) [file pone.0248294.s012.docx]

## S2 File. DNA barcoding analysis details.

### PCR

Amplification reactions were carried out in 12.5 μl volumes and contained 1x PCR buffer (Invitrogen, USA), 200mM each dNTP, 0.2 μM of each primer, 0.8 mg/ml of BSA, and 0.5 units of AmpliTaq Gold™ polymerase (Invitrogen, USA). Samples were first denatured for 10 minutes at 95° C followed by 35 cycles of 95° C for 30 sec, 55/58° C for 30 sec and 72°C for 30 sec and a final extension step of 72°C for five minutes. The indexing PCR step was carried out in 50 μl volumes with the same concentration of reagents as per the initial PCR with the exception of BSA, which was not included. Cycling conditions were an initial denaturation of three minutes at 95° C followed by 15 cycles of 98° C for 20 sec, 56° C for 30 sec and 72°C for 30 sec and a final extension step of 72°C for five minutes.

### Library purification and pooling

PCR products from the indexing step were purified following the bead clean-up protocol in (Rohland and Reich 2012) modified such that 1% of Sera-Magnetic beads were used rather than 0.5%. Elution was performed with 20 μl of Buffer AL (Qiagen, Germany). Concentrations of individual (for the 3 different primer sets) PCR products was determined with the *Quantifluor® dsDNA* system (Promega, USA) on a 96-well microplate Fluorometer (Twinkle LB 970, Berthold, USA; emission: 535 nm, excitation: 485 nm). Standards were measured in triplicate (5-fold serial dilution, 250 ng/ml – 0.2 ng/ml) and samples in duplicate and subsequently average values calculated. Samples were then pooled in equimolar concentrations.
