# Supplementary material for: Isotope analysis combined with DNA barcoding provide new insights into the dietary niche of khulan in the Mongolian Gobi
Source: PLoS One. 2021 Mar 29;16(3):e0248294. doi: 10.1371/journal.pone.0248294 (PMC8006982; doi:10.1371/journal.pone.0248294)
Supplement: S3 File — (ZIP) [file pone.0248294.s013.zip › Khulan_diet_scripts/Metabarcoding_pipeline.docx]

**Meta-barcoding Pipeline**

Starting with demultiplexed raw .bam files

|  | **Step** | **Program** | **Script/Command** |
| --- | --- | --- | --- |
| 1. | Convert to fastq | Samtools | convert_bam2fq.sh |
| 2. | Merge paired-ends | FLASH | merge.sh |
| 3. | Trim adaptors x 2 | Flexbar | trim_adaptor.sh |
| 4. | Quality Filter | Fastx | qc_filt.sh |
| 5. | Pad reads / trim | Usearch | pad_trim.sh |
| 6. | Re-label reads / convert to fasta | Usearch | label_conv.sh |
| 7. | Pool samples | Command line | “cat *.fasta > Pool.fa” |
| 8. | Format fasta file | Fastx | “fasta_formatter –i infile –o outfile” |
| 9. | De-replicate Pool | Fastx | “fastx_collapser –i infile –o outfile” |
| 10. | Add size label | Command line | “sed -i 's/-/;size=/g' file.txt” |
| 11. | Cluster OTU’s | Usearch | “cluster_otus” |
| 12. | Assign taxonomy to OTU’s | CLC workbench | Blast NCBI |
| 13. | De-replicate Samples | Usearch | derep_label.sh |
| 14. | Pool De-replicated Samples | Command line | “cat *sc.fasta > Pool_sc.fa” |
| 15. | Format sample names |  | sed -i 's\/ReadX\/>ReadX_/g' Pool_sc.fa |
| 16. | Make OTU Table | Usearch | “-otutab Pool_ sc.fa -otus file_otus -otutabout diet_analysis_otutab1.txt -strand both” |
