# Supplementary figures and images for: Isotope analysis combined with DNA barcoding provide new insights into the dietary niche of khulan in the Mongolian Gobi
Source: PLoS One. 2021 Mar 29;16(3):e0248294. doi: 10.1371/journal.pone.0248294 (PMC8006982; doi:10.1371/journal.pone.0248294)

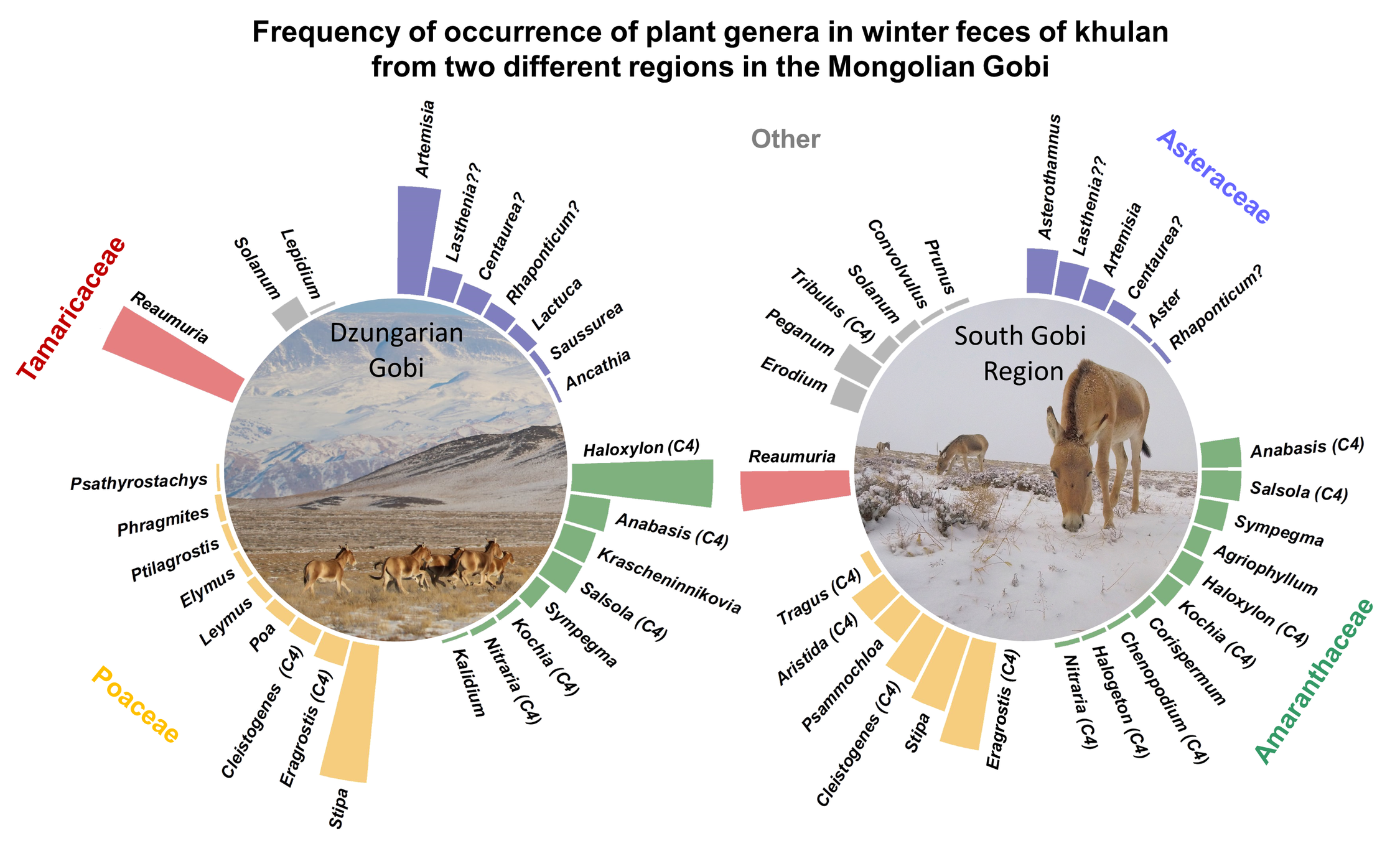

Supplement: S1 Graphical abstract — (TIF) [file pone.0248294.s014.tif]
